# Predicting three-month fasting blood glucose and glycated hemoglobin changes in patients with type 2 diabetes mellitus based on multiple machine learning algorithms

**DOI:** 10.1038/s41598-023-43240-5

**Published:** 2023-09-30

**Authors:** Xue Tao, Min Jiang, Yumeng Liu, Qi Hu, Baoqiang Zhu, Jiaqiang Hu, Wenmei Guo, Xingwei Wu, Yu Xiong, Xia Shi, Xueli Zhang, Xu Han, Wenyuan Li, Rongsheng Tong, Enwu Long

**Affiliations:** 1 Personalized Drug Therapy Key Laboratory of Sichuan Province, Department of Pharmacy, Sichuan Provincial People’s Hospital, School of Medicine, University of Electronic Science and Technology of China, Chengdu, 610072 Sichuan China; 2 West China School of Public Health and West China Fourth Hospital, Sichuan University, Chengdu, 610044 Sichuan China; 3 Department of Pharmacy, Daping Hospital, Army Medical University, Chongqing, 400042 China; 4 Hospital of Chengdu University of Traditional Chinese Medicine, Chengdu, 610072 Sichuan China; 5 School of Pharmacy, Southwest Medical University, Luzhou, 646000 Sichuan China; 6 Institute of Materia Medica, Chinese Academy of Medical Sciences/Peking Union Medical College, Beijing, 100050 China; 7 Sichuan Provincial Health Information Center, Chengdu, 610015 Sichuan China

**Keywords:** Endocrinology, Endocrine system and metabolic diseases

## Abstract

Fasting blood glucose (FBG) and glycosylated hemoglobin (HbA1c) are key indicators reflecting blood glucose control in type 2 diabetes mellitus (T2DM) patients. The purpose of this study is to establish a predictive model for blood glucose changes in T2DM patients after 3 months of treatment, achieving personalized treatment.A retrospective study was conducted on type 2 diabetes mellitus real-world medical data from 4 cities in Sichuan Province, China from January 2015 to December 2020. After data preprocessing, data inputting, data sampling, and feature screening, 16 kinds of machine learning methods were used to construct prediction models, and 5 prediction models with the best prediction performance were screened respectively. A total of 100,000 cases were included to establish the FBG model, and 2,169 cases were established to establish the HbA1c model. The best prediction model both of FBG and HbA1c finally obtained are realized by ensemble learning and modified random forest inputting, the AUC values are 0.819 and 0.970, respectively. The most important indicators of the FBG and HbA1c prediction model were FBG and HbA1c. Medication compliance, follow-up outcome, dietary habits, BMI, and waist circumference also had a greater impact on FBG levels. The prediction accuracy of the models of the two blood glucose control indicators is high and has certain clinical applicability.HbA1c and FBG are mutually important predictors, and there is a close relationship between them.

## Introduction

Diabetes mellitus is a chronic progressive disease characterized by disorders of glucose metabolism^1^. In recent years, with the social and economic development of countries around the world and the improvement of residents' living standards, the incidence rate and prevalence rate of diabetes have increased year by year, which has become a major social problem threatening people's health and has attracted the attention and attention of governments, health departments and medical workers in various countries. According to epidemiological data from the International Diabetes Federation (IDF): the global diabetes prevalence in 20–79 years old in 2021 was estimated to be 10.5% (536.6 million people), rising to 12.2% (783.2 million) in 20,452^2^. And in 2021, almost one in two adults (20–79 years old) with diabetes were unaware of their diabetes status (44.7%; 239.7 million)^3^.

Type 2 diabetes mellitus (T2DM) patients accounted for more than 90.0% of the total diabetic patients^4^. With the development of the disease, most T2DM patients will have different degrees of complications, which will seriously reduce the quality of life of the patients and bring a heavy economic burden to the patients' families^5^. And the severity of complications is inseparable from glycemic control. Therefore, active, safe, and effective blood glucose control has positive significance for preventing complications, improving the quality of life of T2DM patients, and reducing the economic burden on patients and society. Fasting blood glucose (FBG) and glycosylated hemoglobin (HbA1c) are the key indicators for clinical diagnosis and evaluation of treatment effect of T2DM. They are used to detect blood glucose and both are important indicators for diagnosing diabetes and reflecting the prognosis of diabetes^6^.

With the continuous development of database and data mining technology, data mining is more and more used to mine medical databases efficiently^7^. The existing data mining technology application research shows that the model established by data mining has high accuracy^8^. There are many researches on building prediction models based on data mining technology to predict diabetes and its complications, so as to prevent diabetes^9,10^. However, in addition to the prediction of diabetes, the control of blood glucose after treatment is also a matter of concern. The improvement of blood glucose in T2DM patients is related to individual differences, because the factors affecting the treatment results of T2DM involve physiology, pathology, diet structure, lifestyle and other aspects. Clinicians need to give individualized treatment plan according to the patient's own situation to ensure the best effect^11,12^.

At present, prediction models based on machine learning algorithms are mainly used for the prediction of diabetes and its complications^13,14^, and there are few studies on the prediction models of patients' glycemic control after medication. Therefore, it is urgent to establish an efficient, accurate and economical prediction model of T2DM treatment results, and improve the treatment rate of T2DM in medical institutions at all levels. Based on this, this study intends to establish artificial intelligence prediction models for the compliance of two blood glucose indicators in T2DM patients after 3 months of treatment through data mining, to explore potential predictive relationships between FBG and HbA1c, improve the treatment rate and control rate of T2DM, reduce the incidence of adverse reactions, and prevent and reduce the occurrence of complications.

## Materials and methods

### Study design and data source

The data of this study were obtained from the Public Health Service System and the Medical Record Homepage Management System of the Health Information Center of Sichuan Province, China (including personal basic information form, health check-up form, and follow-up service record form), and the overall data were derived from patients who received anti-diabetic drugs or had the International Classification of Diseases Tenth Revision (ICD-10) code^15^ for type 2 diabetes between January 2015 and December 2020.

A total of 375,723 T2DM patients' related diagnosis and treatment data were collected in this study, and the available data for constructing the FBG prediction model and the HbA1c prediction model were screened according to the following criteria: if the same patient had 2 or more registration data within 3 ± 1 months, the patient's data was available; If there were multiple sets of data within 3 ± 1 months, the data closest to 3 months from the baseline should be taken; if there were multiple sets of consistent data longitudinally for the same patient (a patient had multiple sets of data that met the requirements of having 2 or more data within 3 ± 1 months), a group was randomly selected for inclusion. Determination of glycemic control status: to facilitate the development of a predictive model, this study converted the continuous measurements of FBG and HbA1c into discrete categorical outcomes. According to the relevant guidelines^16^, the FBG threshold range was defined as [4.4–7.0]. A value of 1 was assigned to patients whose FBG values fell within the well-regulated range (FBG: 4.4–7.0), while a value of 0 was assigned to those outside this range. Similarly, the HbA1c threshold was set at 7%. A value of 1 was attributed to patients who achieved controlled HbA1c levels (HbA1c < 7), and a value of 0 was assigned to those who did not achieve the desired HbA1c control (HbA1c ≥ 7).

The data included the patient's basic information, drug use, test indicators, and living and diet, as well as the actual follow-up of the patient after treatment. This study used a unique ID to identify patient connection information, and all research operations carried out would not be traced to the individual patient, and the patient's sensitive personal information (such as name, phone number, address, work unit, responsible doctor, etc.) would be deleted. All files were encrypted during transmission and use, and documents were received by a password. This study has passed the ethical review, the approval document in Supplementary Fig. 1.

In this study, a total of 511 variables were included, which were named X1–X511 for statistical convenience (Detailed variables are shown in Supplemental Table 1). Data analysis was performed using named variables, and the variable names were restored after the model evaluation process was complete.

**Table 1:** Baseline characteristics of participants.

Predictors	FBG(N = 100,000)	HbA1c(N = 2,169)
Categorical variables, n(%)		
Gender		
Male	38,205 (38.2)	735(33.9)
Female	61,795 (61.8)	1434(66.1)
Education		
College and above	71,532 (71.5)	1631(75.2)
Below college and other	28,648 (28.5)	538(24.8)
Marital status		
Married/living as married/civil partnershi	84,413 (84.5)	1749(80.6)
Single/never marrie	13,867 (13.9)	339(15.6)
Widowed	981 (1.0)	28(1.3)
Divorced or separate	687 (0.6)	6(0.3)
Complication		
Complicated with diabetes-related complications	16,016 (16.0)	820(37.8)
Without diabetes-related complications	83,984 (84.0)	1349(62.2)
Comorbidity		
Cerebrovascular disease	11,018 (11.0)	916(42.2)
Kidney disease	15,752 (15.8)	262(12.1)
Heart disease	13,298 (13.3)	889(41.0)
Vascular disease	11,542 (11.5)	917(42.3)
Ophthalmological disease	11,155 (11.2)	897(41.4)
Hypertensio	54,363 (54.4)	1354(62.4)
Drug		
Metformin	16,978 (17.0)	–
Grezit	8,421 (15.7)	–
Continuous variables, mean (SD)		
FBG, mmol/L	8.6 (3.9)	8.9(3.8)
HbA1c, %	7.8 (2.9)	7.8(2.9)
Pulse rate, CPM	75.3 (10.6)	76(10.4)
BMI, kg/m	24.8 (3.6)	25.4(3.7)
Waist circumference, cm	84.3 (9.2)	85.6(9.2)
Hemoglobin, g/L	133.9 (17.9)	135.1(15.9)
Leukocyte, × 10^9^ /L	6.5 (3.2)	6.5(1.8)
Platelet, × 10^9^ /L	178.1 (67.8)	189.7(63.6)
ALT, U/L	25.3 (16.2)	24.7(16.3)
AST, U/L	24.1 (14.2)	20.8(14.2)
Albumin, g/L	39.5 (13.4)	–
Total bilirubin, μmol/L	13.6 (10.7)	13.2(6.3)
Conjugated bilirubin, μmol/L	4.5 (2.9)	–
Scr, μmol/L	75.6 (33.6)	62.8(29.9)
Blood urea nitrogen, mmol/L	5.7 (2.2)	5.7(1.9)
TC, mmol/L	4.7 (1.5)	4.8(1.8)
TG, mmol/L	1.9 (2.4)	2.2(2.5)
DBP, mmHg	80.1 (9.4)	80.2(8.9)
SBP, mmHg	134.9 (16.4)	134.3(15.6)

n (%), number of patients and percentage over the total number of patients; mean(SD), the mean and standard deviation of the variable.

### Data cleaning

We deleted variables with a missing ratio of 90%, a single category ratio of 90%, and variables with a coefficient of variation less than 0.1. These variables had little impact on the establishment of the model, and the analysis was meaningless, so they were deleted. Two methods were used for inputting missing data: not inputting and modified random forest inputting . After the data were inputted, if there was a large difference between the positive and negative sample sizes, the data was balanced by sampling. And we modified outliers to the maximum or minimum value of the norm.

The method of “not inputting” was to delete the missing columns and the missing rows in the data in turn, and finally, we got the data without missing values. The “modified random forest inputting” meant that by continuously introducing the inputted columns into the model, as the amount of data continued to accumulate, the obtained values had a higher accuracy rate, which could achieve a more accurate prediction of missing values.

After the data were inputted, the data were divided into training and test sets for machine learning. And the number of training sets accounted for 80% of the total sample size, and the number of test sets accounted for 20% of the total sample size.

### Feature screening

The data were screened using three methods: Not screening, Lasso screening, and Boruta screening. Feature screening is an important aspect of model building, which helps to exclude relevant variables, biases, and limitations of unnecessary noise, making the final analysis results closer to reality. Lasso are a useful atheoretical approach for both developing predictive models and selecting key indicators within an often substantially larger pool of available indicators by inputting all latent variables at the same time, reducing bias caused by unimportant variables, and selecting only the most important variables from a potentially large initial pool^17^. Boruta screening is also a popular method at present^18^. It uses the random forest algorithm to extract feature variables, disrupt the sequence of feature variables, and calculate the importance of feature variables^19^.

### Model training

16 kinds of machine learning algorithms were used for model training, and the data after feature screening were modeled respectively. The specific machine learning algorithm models used included: Logistic regression, Decision Tree, Random Forest, Extra Tree, Stochastic Gradient Descent (SGD), Gaussian Naive Bayes, Bernoulli Naive Bayes, Multinomial Naive Bayes, Quadratic Discriminant Analysis (QDA), Linear Discriminant Analysis (LDA), Passive Aggressive, AdaBoost, Bagging, Gradient Boosting, XGBoost, and Ensemble Learning (The introduction and comparison of various machine learning algorithms are detailed in the references^20,21^). In 2011, Tianqi Chen and Carlos Guestrin first proposed the XGBoost algorithm. It is a machine learning model that achieves stronger learning effects by integrating multiple weak learners^22^, and has better flexibility and scalability. Compared with general machine learning algorithms, the XGBoost model shows strong advantages. These machine learning algorithms have their strengths, among which, the ensemble learning model is an evaluation index based on the trained model, summarizing the best model and outputting according to the voting principle. The evaluation indicators of the prediction model included Area Under Curve (AUC), Accuracy, Precision, Recall, and F1 Score. According to the machine learning results, the 5 models with the best prediction performance were selected and their receiver operating characteristic curve (ROC) and P-R curves were drawn.

### Model verification

Ten-fold cross-validation and bootstrapping sampling were used to verify the impact of different preprocessing algorithms and different machine learning algorithms on the prediction of building FBG and HbAlc models^23^. The model with the largest AUC was selected and constructed using 10 subsets (randomly drawn 10%–100% of the total sample size) to assess the effect of different sample sizes on predictive power. Each subset was split 4:1 into a training set and a test set, and the AUC calculated from the test set was used for sample size checking. By transforming randomly sampled data, 10 independent replicates were generated for each model.

**Figure 1:**
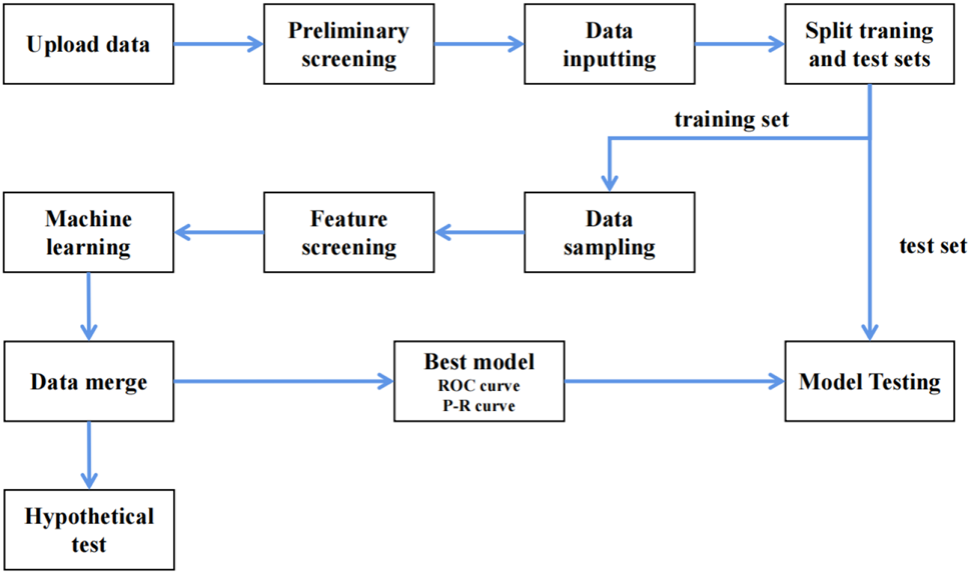
Data analysis flow chart.

A process framework of the data flow is shown in Fig. 1. Data flowed through each node according to a predetermined schedule.

### Statistical analysis

Continuous variables were expressed as mean ± standard deviation, and count variables were expressed as frequency. Differences between quantitative data were tested using a t-test and rank test. Hypothesis testing was used to investigate the influence of different data processing methods and algorithms on the model prediction performance. On the analysis results of bootstrapping sampling and validation set, hypothesis testing single factor analysis was performed. The analysis content included different data inputting methods, feature screening methods, and the corresponding mean ± standard deviation and 95% confidence interval between the three dimensions of the machine learning model and the five evaluation indicators (AUC, Accuracy, Precision, Recall, and F1 Score) and p-value.

Excel 2016 was used for summarizing data, and all statistical analyses were performed using Python 3.8.

The transparent reporting of a multivariable prediction model for individual prognosis or diagnosis (TRIPOD) statement for prediction model development is in Supplemental Table 10.

## Results

### Baseline characteristics

The FBG study cohort included 100,000 patients and the HbA1c study cohort included 2,169 patients. In the FBG study cohort, the sample sizes assigned to 1 and 0 are 50,000, respectively, and in the HbAlc study cohort, the sample sizes assigned to 1 and 0 are 1,027 and 1,142, respectively. Baseline demographic, clinical, laboratory and medication details are shown in Table 1. The mean ages of the two cohorts were 64.0 ± 10.1 years and 63.1 ± 10.2 years, respectively. The most common comorbidities in both cohorts were hypertension, kidney disease, and heart disease. At baseline, FBG was 8.6 ± 3.9 mmol/L and 8.9 ± 3.8 mmol/L, respectively, and HbA1c were both 7.8 ± 2.8%.

### Variable and feature screening

426 and 432 variables were removed from the FBG prediction model and the HbA1c prediction model, respectively, during data cleaning, and the specific variables are shown in Supplementary Table 2. Therefore, a total of 85 and 79 variables were finally used for modeling, respectively (Supplementary Table. 3). After inputting data with missing variables, the positive and negative samples are relatively balanced, so no sampling is required.

**Figure 2:**
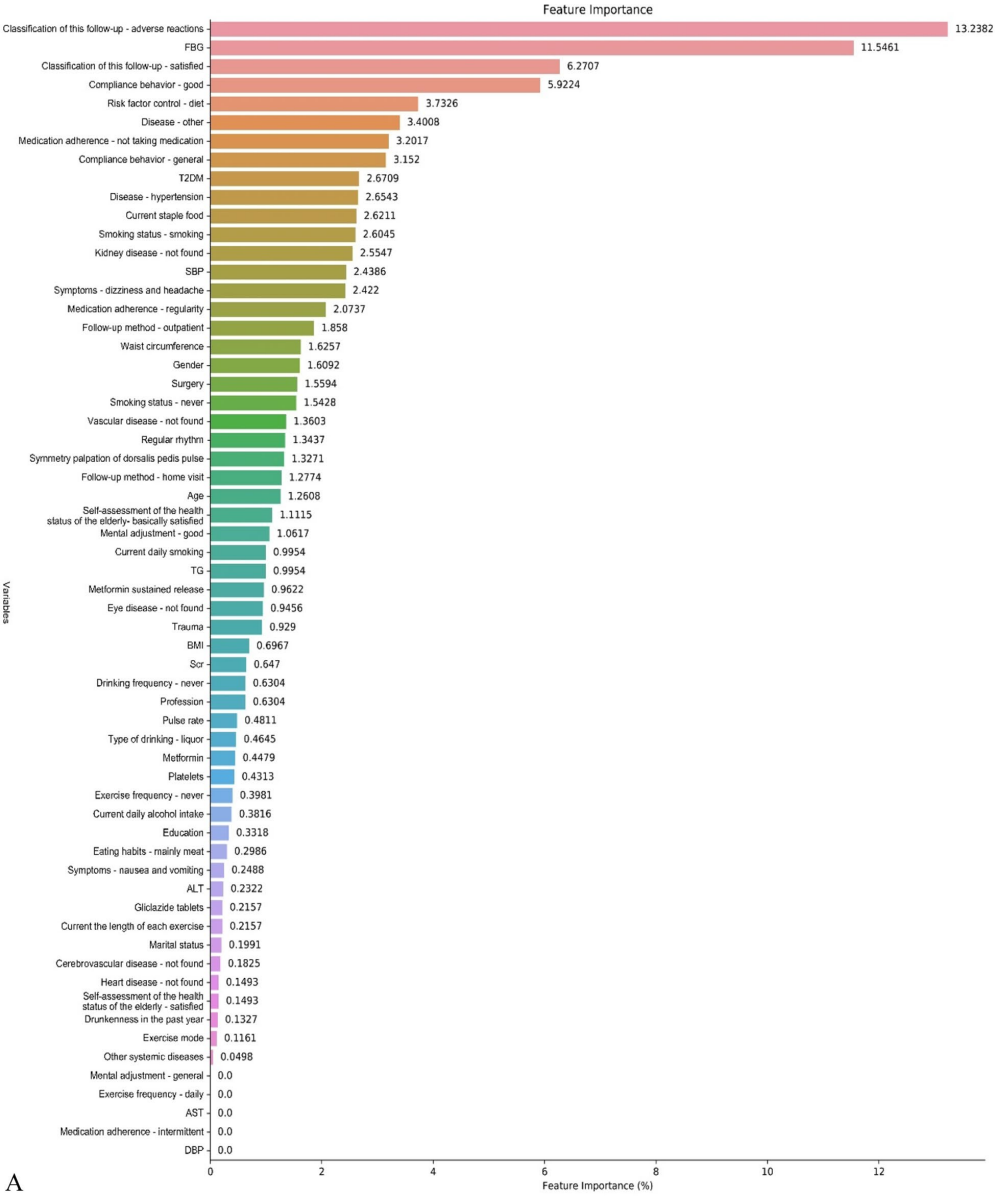

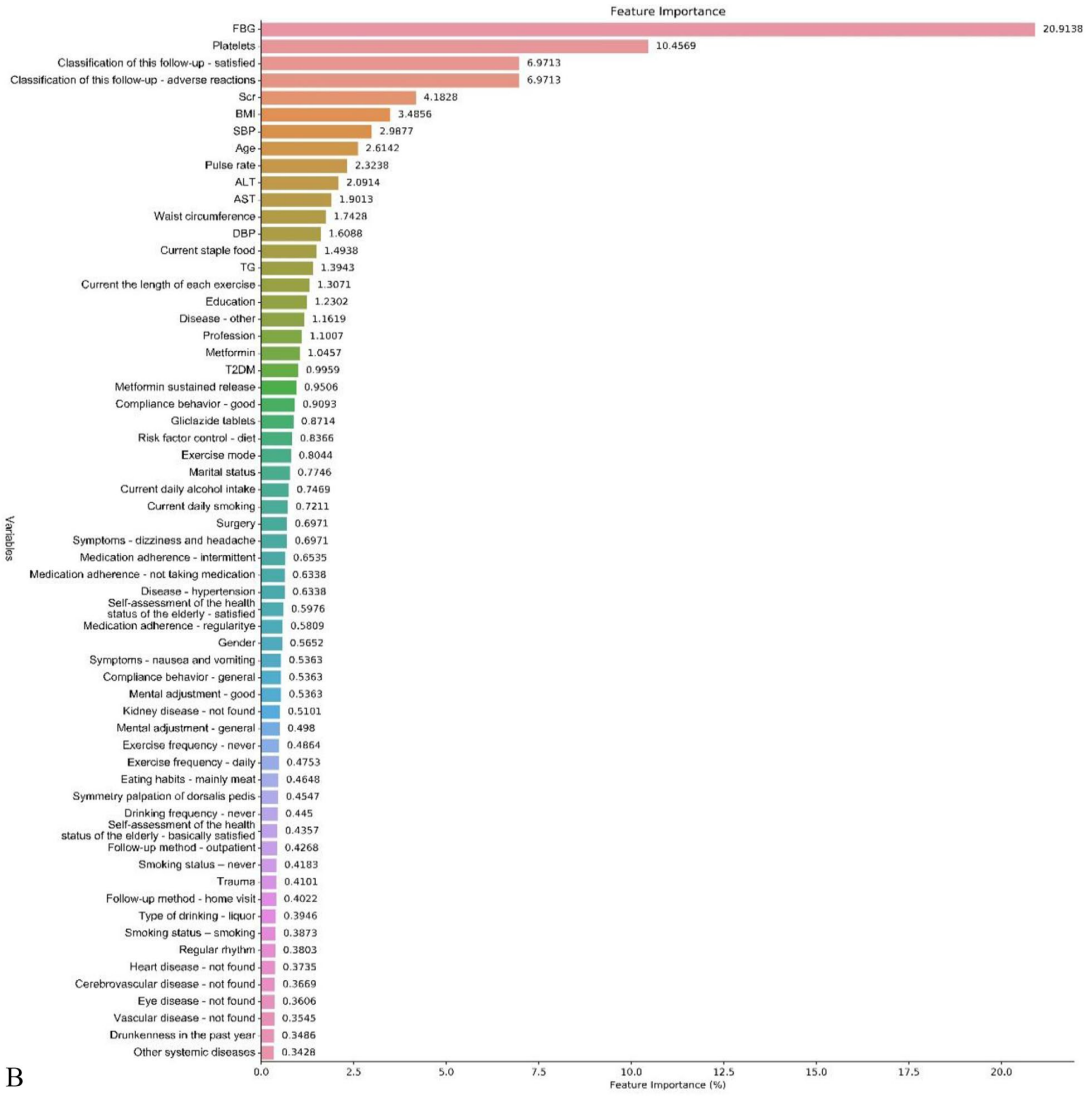
Feature importance bar chart—**(A)** FBG; (**B)** HbA1c (inputting method: not; screening method: lasso screening).

The results of the feature screening are shown in Table 2. The results showed that the most important indicators of the FBG and HbA1c prediction model were the FBG value and HbA1c. The patient's medication compliance, follow-up, dietary habits, BMI, and waist circumference also had a greater impact on the FBG level. In addition, the feature selection results also showed that patients with hypertension or other comorbid diseases, laboratory indicators of related diseases such as Scr, serum alanine aminotransferase, blood urea nitrogen, platelets, and white blood cells, as well as age, smoking, all had a certain degree of influence on FBG. For the HbA1c prediction model, the feature screening results showed that laboratory indicators such as platelets, Scr, AST, Hb, AST, etc. accounted for a large proportion. According to the results of Not inputting and Lasso screening, the feature importance of the data is drawn (Fig. 2), and the feature importance bar chart drawn by other inputting and screening methods is shown in supplementary Figs. 2–7.

**Table 2:** Feature screening results.

Inputting method	Screening method	Top 10 variables	
		FBG	HbA1c
Not	Lasso	Adverse reactions, FBG, Satisfaction with follow-up, Medical Compliance—Good, diet control, Concomitant disease, Medication Adherence—Not Taking Medication, Medical Compliance—general, T2DM, hypertension	FBG, Platelets, Satisfaction with follow-up, Adverse reactions, Scr, BMI, SBP, Age, Pulse rate, ALT, AST
Not	Boruta	FBG、platelets, Satisfaction with follow-up, Adverse reactions, Scr, BMI, high blood pressure, age, Pulse rate, ALT	FBG, pulse rate, Scr, HBP, BMI, SBP, Waist circumference, AST, Age, daily staple food
Modified random forest	Lasso	HbA1c, Adverse reactions, Satisfaction with follow-up, Medical Compliance—Good, FBG, diet control, Medication Adherence—Not Taking Medication, Concomitant disease, Outpatient follow-up, kidney disease	HbA1c, Age, Medication Adherence—Not Taking, Adverse reactions , Symmetry palpation of dorsalis pedis, pulse rate, SBP, FBG, Current the length of each exercise, BMI
Modified random forest	Boruta	Platelets, Adverse reactions, FBG, BMI, blood urea nitrogen, HbA1c, leukocyte, Satisfaction with follow-up, high blood pressure, Scr	Age, Hb, HBP, BUN, Scr, AST, HbA1c, PLT, FBG, BMI

### Model performance

**Figure 3:**
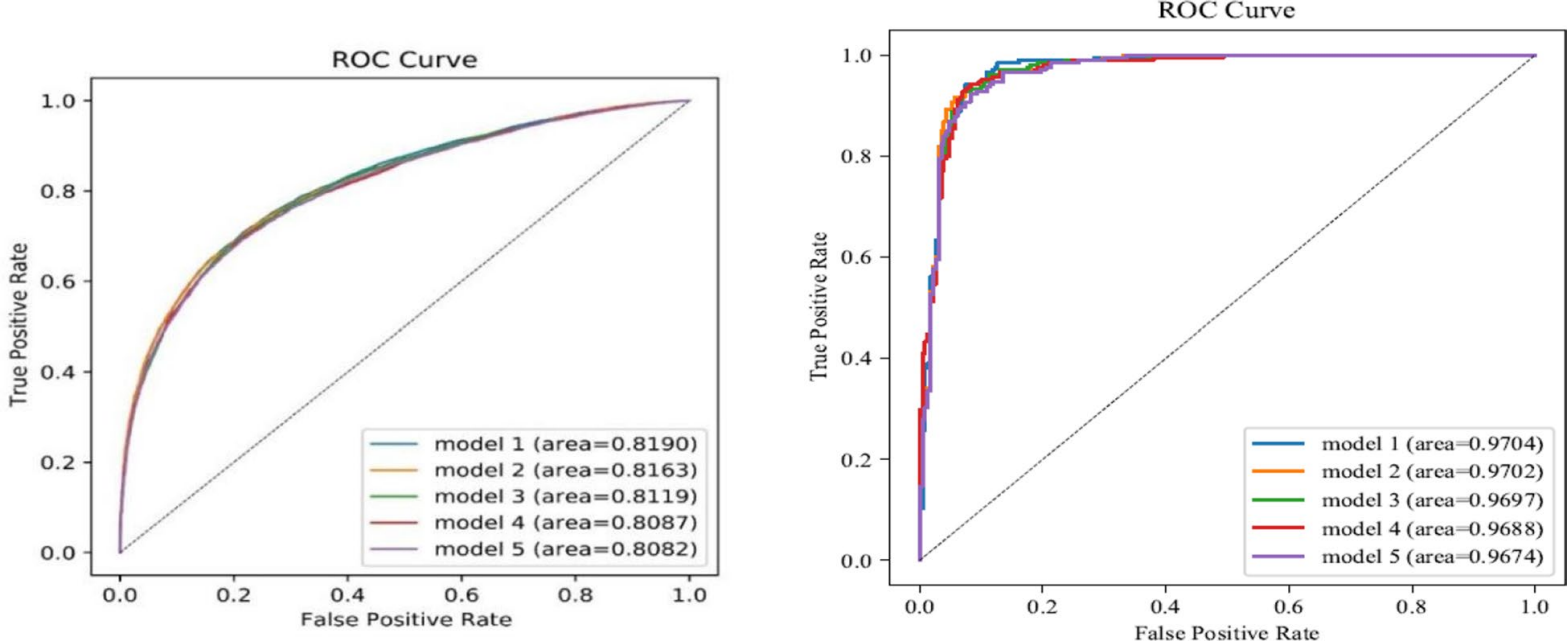
ROC curves of the five best predictive models (**A** FBG; **B** HbA1c).

**Figure 4:**
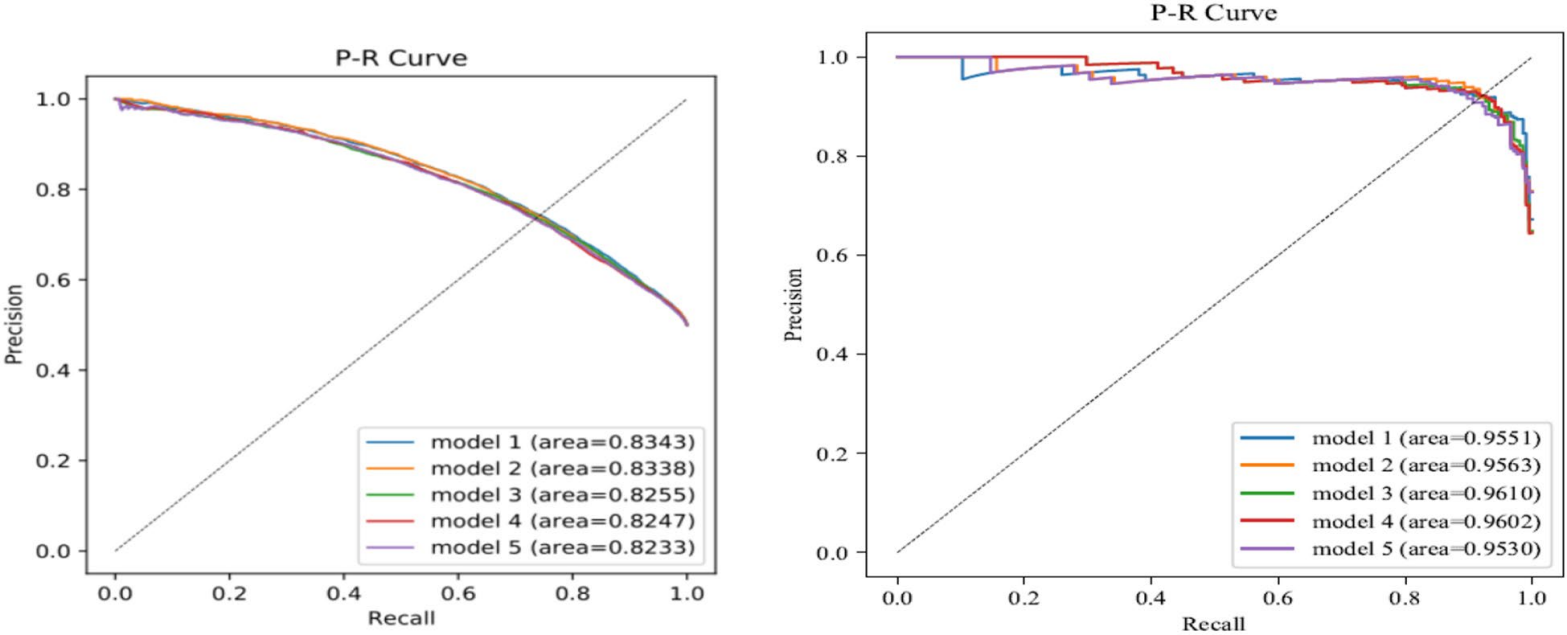
P-R curves of the five best predictive models(**A** FBG; **B** HbA1c).

The machine learning results are shown in Tables 3 and 4. The inputting methods used by all best models were all modified random forest inputting: The optimal feature screening method was Not screening and Boruta screening The optimal machine learning methods were ensemble learning and XGBoost. The AUC value of the best model for FBG was model 1 (AUC = 0.8190); the worse one was model 5 (AUC = 0.8082). The AUC value of the best model for HbA1c was model 1 (AUC = 0.9704); the worse one was model 5 (AUC = 0.9674). The AUC values of the ten best models were all greater than 0.75, indicating that the prediction model had good prediction performance, and had the possibility of certain clinical application. The ROC curves and P-R curves of the five best models are shown in Figs. 3 and 4.

**Table 3:** Predictive model building results.

	Model ID	AUC	Accuracy	Precision	Recall	F1 score	Inputting methods	Screening methods	Models
FBG	Model 1	0.819	0.7439	0.7733	0.6901	0.7293	Modified random forest inputting	Not	Ensemble learning
	Model 2	0.8163	0.7423	0.7674	0.6955	0.7297	Modified random forest inputting	Not	XGBoost
	Model 3	0.8119	0.7415	0.7692	0.69	0.7275	Modified random forest inputting	Boruta	Ensemble learning
	Model 4	0.8087	0.7404	0.769	0.6872	0.7258	Modified random forest inputting	Lasso	Ensemble learning
	Model 5	0.8082	0.7388	0.7629	0.6929	0.7262	Modified random forest inputting	Boruta	XGBoost
HbA1c	Model 1	0.9704	0.9217	0.894	0.9463	0.9194	Modified random forest inputting	Boruta	Ensemble learning
	Model 2	0.9702	0.924	0.9135	0.9268	0.9201	Modified random forest inputting	Not	Ensemble learning
	Model 3	0.9697	0.924	0.9095	0.9317	0.9205	Modified random forest inputting	Lasso	Ensemble learning
	Model 4	0.9688	0.9263	0.9179	0.9268	0.9223	Modified random forest inputting	Lasso	XGBoost
	Model 5	0.9674	0.9171	0.9043	0.922	0.913	Modified random forest inputting	Not	XGBoost

**Table 4:** Machine learning algorithm ten-fold cross-validation analysis results.

FBG	AUC		Accuracy		Precision		Recall		F1Score	
	Mean ± SD	95%CI	Mean ± SD	95%CI	Mean ± SD	95%CI	Mean ± SD	95%CI	Mean ± SD	95%CI
AdaBoost	0.741 ± 0.015	0.738–0.744	0.682 ± 0.012	0.680–0.684	0.679 ± 0.019	0.675–0.682	0.677 ± 0.020	0.674–0.681	0.678 ± 0.016	0.675–0.681
Bagging	0.747 ± 0.033	0.741–0.753	0.690 ± 0.026	0.685–0.695	0.694 ± 0.038	0.687–0.701	0.668 ± 0.026	0.664–0.673	0.681 ± 0.029	0.675–0.686
Bernoulli_Naive_Bayes	0.722 ± 0.010	0.721–0.724	0.673 ± 0.008	0.672–0.675	0.663 ± 0.009	0.661–0.665	0.688 ± 0.022	0.684–0.692	0.675 ± 0.015	0.673–0.678
Decision_Tree	0.738 ± 0.019	0.735–0.742	0.685 ± 0.015	0.683–0.688	0.689 ± 0.037	0.682–0.695	0.671 ± 0.038	0.664–0.678	0.678 ± 0.015	0.675–0.681
Extra_Tree	0.724 ± 0.017	0.721–0.727	0.677 ± 0.013	0.674–0.679	0.677 ± 0.025	0.673–0.682	0.663 ± 0.028	0.658–0.668	0.669 ± 0.016	0.667–0.672
Gaussian_Naive_Bayes	0.696 ± 0.014	0.693–0.698	0.647 ± 0.012	0.645–0.649	0.630 ± 0.010	0.628–0.631	0.694 ± 0.030	0.689–0.700	0.660 ± 0.017	0.657–0.663
Gradient_Boosting	0.750 ± 0.018	0.747–0.753	0.690 ± 0.015	0.687–0.692	0.695 ± 0.029	0.689–0.700	0.666 ± 0.017	0.663–0.669	0.680 ± 0.015	0.677–0.682
LDA	0.737 ± 0.012	0.734–0.739	0.678 ± 0.007	0.676–0.679	0.668 ± 0.007	0.666–0.669	0.692 ± 0.028	0.687–0.697	0.679 ± 0.016	0.676–0.682
Logistic_Regression	0.737 ± 0.012	0.735–0.740	0.679 ± 0.008	0.677–0.680	0.670 ± 0.008	0.669–0.672	0.688 ± 0.029	0.683–0.693	0.679 ± 0.017	0.676–0.682
Multinomial_Naive_Bayes	0.707 ± 0.009	0.705–0.708	0.666 ± 0.006	0.665–0.667	0.657 ± 0.006	0.656–0.658	0.678 ± 0.035	0.672–0.684	0.667 ± 0.017	0.664–0.670
Passive_Aggressive	0.610 ± 0.052	0.600–0.619	0.581 ± 0.040	0.574–0.588	0.575 ± 0.044	0.567–0.583	0.579 ± 0.076	0.565–0.593	0.575 ± 0.054	0.565–0.585
QDA	0.723 ± 0.010	0.721–0.725	0.670 ± 0.008	0.669–0.672	0.660 ± 0.008	0.659–0.662	0.686 ± 0.023	0.682–0.690	0.673 ± 0.015	0.670–0.675
Random_Forest	0.756 ± 0.027	0.751–0.761	0.696 ± 0.022	0.692–0.700	0.702 ± 0.033	0.696–0.708	0.669 ± 0.025	0.664–0.674	0.685 ± 0.025	0.680–0.690
SGD	0.736 ± 0.012	0.734–0.739	0.670 ± 0.005	0.669–0.670	0.652 ± 0.004	0.651–0.652	0.712 ± 0.034	0.706–0.718	0.680 ± 0.016	0.677–0.683
XGBoost	0.761 ± 0.029	0.756–0.766	0.698 ± 0.023	0.694–0.702	0.702 ± 0.035	0.696–0.708	0.679 ± 0.021	0.675–0.683	0.690 ± 0.024	0.686–0.694
P value	P < 0.0001		P < 0.0001		P < 0.0001		P < 0.0001		P < 0.0001	
HbA1c										
AdaBoost	0.782 ± 0.141	0.756–0.807	0.737 ± 0.120	0.716–0.759	0.762 ± 0.130	0.738–0.786	0.737 ± 0.150	0.710–0.764	0.739 ± 0.111	0.719–0.759
Bagging	0.796 ± 0.161	0.767–0.825	0.747 ± 0.143	0.721–0.772	0.763 ± 0.131	0.739–0.786	0.741 ± 0.145	0.715–0.767	0.749 ± 0.133	0.725–0.773
Bernoulli_Naive_Bayes	0.727 ± 0.103	0.708–0.745	0.682 ± 0.101	0.664–0.701	0.683 ± 0.093	0.666–0.700	0.692 ± 0.123	0.670–0.715	0.685 ± 0.100	0.667–0.703
Decision_Tree	0.773 ± 0.142	0.747–0.799	0.742 ± 0.125	0.719–0.764	0.750 ± 0.133	0.726–0.774	0.773 ± 0.101	0.755–0.791	0.756 ± 0.101	0.738–0.775
Extra_Tree	0.740 ± 0.124	0.718–0.763	0.710 ± 0.104	0.691–0.729	0.718 ± 0.116	0.697–0.739	0.740 ± 0.140	0.715–0.765	0.719 ± 0.095	0.702–0.736
Gaussian_Naive_Bayes	0.699 ± 0.114	0.679–0.720	0.606 ± 0.114	0.585–0.626	0.602 ± 0.108	0.582–0.622	0.808 ± 0.176	0.776–0.840	0.671 ± 0.090	0.655–0.687
Gradient_Boosting	0.801 ± 0.151	0.774–0.829	0.747 ± 0.135	0.723–0.772	0.765 ± 0.131	0.742–0.789	0.754 ± 0.130	0.731–0.778	0.755 ± 0.117	0.734–0.776
LDA	0.766 ± 0.132	0.742–0.790	0.716 ± 0.118	0.695–0.737	0.715 ± 0.103	0.696–0.734	0.743 ± 0.126	0.720–0.765	0.726 ± 0.106	0.707–0.745
Logistic_Regression	0.779 ± 0.144	0.753–0.805	0.743 ± 0.129	0.720–0.767	0.749 ± 0.120	0.727–0.771	0.761 ± 0.138	0.736–0.786	0.751 ± 0.118	0.730–0.773
Multinomial_Naive_Bayes	0.662 ± 0.076	0.648–0.676	0.603 ± 0.054	0.593–0.613	0.602 ± 0.051	0.592–0.611	0.613 ± 0.161	0.584–0.642	0.599 ± 0.086	0.583–0.614
Passive_Aggressive	0.694 ± 0.148	0.668–0.721	0.649 ± 0.131	0.625–0.672	0.646 ± 0.148	0.620–0.673	0.648 ± 0.186	0.615–0.682	0.641 ± 0.156	0.612–0.669
QDA	0.730 ± 0.111	0.710–0.750	0.680 ± 0.100	0.662–0.698	0.674 ± 0.088	0.658–0.690	0.719 ± 0.109	0.699–0.738	0.694 ± 0.090	0.677–0.710
Random_Forest	0.791 ± 0.152	0.763–0.818	0.740 ± 0.135	0.715–0.764	0.762 ± 0.131	0.738–0.786	0.731 ± 0.129	0.708–0.754	0.743 ± 0.121	0.721–0.765
SGD	0.785 ± 0.140	0.760–0.810	0.740 ± 0.132	0.716–0.764	0.744 ± 0.125	0.721–0.767	0.767 ± 0.127	0.745–0.790	0.752 ± 0.116	0.731–0.773
XGBoost	0.802 ± 0.159	0.773–0.831	0.753 ± 0.146	0.727–0.779	0.768 ± 0.138	0.743–0.793	0.754 ± 0.143	0.728–0.780	0.758 ± 0.134	0.734–0.782
P value	P < 0.0001		P < 0.0001		P < 0.0001		P < 0.0001		P < 0.0001

### The effect of different data processing methods on the results

The results of the ten-fold cross-validation analysis of the data inputting method for FBG are shown in Supplemental Table 4. The modified random forest inputting had the greater impact on the model effect, the AUC values for the FBG and HbA1c models were 0.749 ± 0.044 (95%CI = 0.745–0.753) and 0.901 ± 0.078 (95%CI = 0.894–0.907). The results of the ten-fold cross-validation analysis of the feature screening of the validation set showed that (Supplemental Table 5), Lasso screening had the greatest impact on the model effect, the AUC values for the FBG, and HbA1c models were 0.728 ± 0.038 (95%CI = 0.725–0.731) and 0.776 ± 0.130 (95%CI = 0.766–0.785).

The analysis results of the data inputting method in the bootstrapping sampling showed that the modified random forest inputting had a greater impact on the model effect (Supplemental Table 6), the AUC values for the FBG and HbA1c models were 0.754 ± 0.048 (95%CI = 0.754–0.755) and 0.902 ± 0.083 (95%CI = 0.902–0.903). The results of feature screening analysis in bootstrapping sampling showed that Boruta screening had the greatest impact on the FBG model effect, with an AUC value of 0.732 ± 0.040 (95%CI = 0.731–0.732). For HbA1c, the greatest impact analysis screening method in bootstrapping sampling was Lasso screening, with an AUC value of 0.772 ± 0.131 (95%CI = 0.772–0.773) (Supplemental Table 7).

### The effect of different algorithms on the results

Hypothesis testing was used to examine the impact of different algorithms on the model's predictive performance. The ten-fold cross-validation results for FBG and HbA1c prediction model are shown in Supplemental Table 8. The results showed that XGBoost had the greatest impact on the model effect, the mean AUC values for the FBG and HbA1c models were 0.761 ± 0.029 (95% CI = 0.756–0.766) and 0.802 ± 0.159 (95% CI = 0.773–0.831). The results of bootstrapping sampling analysis (Supplemental Table 9) showed that among the 16 machine learning models used in this section, ensemble learning had the greatest impact on the model effect, the mean AUC values of FBG and HbA1c are respectively 0.767 ± 0.029 and 0.843 ± 0.11.

## Discussion

The factors that affect the treatment results of diabetes are very complex, including age, gender, weight, course of disease, eating habits, lifestyle, organ function, etc. Therefore, the treatment of T2DM needs to consider multiple risk factors according to the actual diagnosis and treatment of patients, and provide appropriate treatment plans for different patients. Effectively evaluating and predicting the improvement of blood glucose after treatment can help clinicians better provide individualized treatment services for patients, while FBG and HbAlc are the most commonly accepted indicators to measure the improvement of blood glucose. Our research is based on machine learning methods and aims to build artificial intelligence prediction models for FBG and HbAlc in patients with T2DM. By analyzing the influencing factors of related blood glucose control indicators in T2DM patients, we established two prediction models for FBG and HbAlc, and they can assist clinical treatment and T2DM patient management, to allow early adjustment of the treatment plan and improve the treatment rate and control rate of T2DM. Our analysis results show that: (1) FBG, HbA1c, medication compliance, and dietary habits have a greater impact on both prediction models; (2) The multi-parameter predictive risk models incorporate variables from different domains, including baseline demographics, complications, and laboratory tests, and can accurately predict three-month FBG values and HbAlc values; (3) In the machine learning-driven algorithm, the optimal models both adopt the ensemble learning algorithm. The influence of different algorithms and data processing methods on the results shows that the algorithms that have the greatest impact on the model effect are ensemble learning and XGBoost, and the best inputting method is the modified random forest inputting. Ensemble learning is an advanced machine learning strategy that can improve classification performance and generalization by combining multiple models^24^. Nemat H et al. utilized deep learning and ensemble learning to predict blood glucose levels, and compared the performance of the proposed ensemble model with the non-ensemble model, and the results showed that the developed ensemble model outperformed the non-ensemble baseline model^25^. In our research, we combine the remaining model indicators that have been trained, and the integrated model indicators have great advantages.

From the feature screening results, this study is consistent with other studies, FBG and HbAlc are both the most important predictors. Del Parigi A et al.^26^ used several machine learning algorithms to find predictors of glycemic control in diabetes and found that HbA1c and FPG were the strongest predictors of achieving glycemic control. This is consistent with our findings. For this result, we explain that current FBG and HbA1c values have important effects on future FBG and HbA1c values, respectively. In our study, FBG and HbAlc were mutually important predictors, indicating an important correlation between glycated hemoglobin and fasting blood glucose. The reason maybe is that once the glucose in human blood combines with hemoglobin to form glycosylated hemoglobin, it will age with the aging of red blood cells, which is the product of an irreversible glycation reaction. The contact time between blood glucose and hemoglobin and the content of blood glucose can determine the level of HbA1c, so the content of HbA1c is positively correlated with the blood glucose content of diabetic patients, which may have the ability to predict each other. Studies have shown that postprandial blood glucose and fasting blood glucose are closely related to glycosylated hemoglobin, and for poorly controlled diabetic patients, the greater the value of HbA1c, the greater the contribution of fasting blood glucose value^27^. Wang J et al^28^ .established a blood glucose prediction model. After feature screening, the top six indicators were: age, fasting glutamate transaminase (ALT), blood urea nitrogen (BUN), total protein (TP), uric acid (UA), and BMI. BMI is also in the top ten important features in this study, and the rest of the indicators did not enter the top ten in importance. However, our study also found that ALT and BUN will have a certain degree of influence on blood glucose. Wang YS et al. established a T2DM prediction model in western Xinjiang, China, and used Lasso screening for feature screening^29^. The study showed that age, family history of T2DM, waist circumference, TC, TG, BMI, HDL-C, and previous history of hypertension had a significant impact on FBG. These factors are included in the feature selection results of our study. Chien KL et al^30^ used multiple logistic regression to predict HbA1c and found that both waist circumference and BMI were associated with abnormal glycated hemoglobin levels. Age, family history of diabetes, systolic blood pressure, and biochemical markers including C-reactive protein and triglycerides were significantly associated with higher glycated hemoglobin levels. In our study, waist circumference and BMI had important effects on HbA1c, as did age and hypertension, but the study did not take into account enough variables, and our accuracy is higher. In addition, our study, based on a large sample of physical examination and follow-up data, found that patients' medication compliance, follow-up conditions, and living habits (including dietary habits and smoking) had a greater impact on blood glucose control. The results better clarify the importance of primary prevention of T2DM, which is to focus on changing environmental factors and lifestyles, reducing calorie intake, maintaining a low-salt, low-sugar, high-fiber diet, quitting smoking, limiting alcohol, and getting daily moderate exercise. At the same time, the results of this study also show the importance of follow-up for secondary and tertiary prevention of T2DM. Pourat N et al^31^ conducted an observational study and found that timely linking behavioral health patients to outpatient follow-up after hospitalization is an effective care transition strategy that may reduce readmission rates. Tong L et al^32^ also concluded that follow-up was associated with a reduced risk of readmission. Patients benefited the most from outpatient follow-up because face-to-face conversations allowed more information (both therapeutic and emotional) to be exchanged with patients and better individualized care for patients. In addition, adverse reaction monitoring during the follow-up process can timely detect the risk of hypoglycemia in patients, which brings greater benefits to patients.

Most studies tend to use machine learning algorithms such as decision trees, random forests, SVM, logistic regression, and neural networks to build T2DM prediction models, with AUC values ranging from 0.7 to 0.9^33,34^. Wang J et al. adopted three commonly used machine learning algorithms (RF, SVM, and BP-ANN) combined with the elastic network (EN) to simulate and predict blood glucose status in China. The AUCs of RF, SVM and BP were 0.75, 0.72 and 0.72, respectively^35^. In a study of T2DM prediction models in Australia by Zhang L et al^36^, the model built using XGBoost had the best prediction ability, with a 3-year prediction model AUC value of 0.78 and a 10-year AUC value of 0.75. Xue M et al^37^ established a T2DM prediction model using algorithms such as decision trees, random forests, AdaBoost with decision trees (AdaBoost), and extreme gradient boosting decision trees (XGBoost), and XGBoost had the best performance (AUC = 0.968). Usually, the AUC value is above 0.8, showing a good classification effect^38^. The AUC values of the five optimal FBG models obtained in our study are all greater than 0.8 and the AUC values of the five optimal HbA1c models obtained in our study are all greater than 0.9. Based on incorporating 100,000 pieces of data, 85 variables, and 16 machine learning algorithms for research, we obtained the FBG prediction model with the best AUC value of 0.819 and the HbA1c prediction model with the best AUC value of 0.970, indicating that these prediction models have better performance and better clinical prediction ability. The establishment of these two models can input the current gap in the prediction model of individualized treatment of T2DM patients, provide new ideas and methods for T2DM treatment, and provide T2DM patients with efficient and accurate individualized treatment plans to solve the real health problems of patients. In addition, this study explores the T2DM prediction model based on real-world medical data mining, uses multiple classifiers for comparative research, and selects the optimal model to ensure the optimization of the model, which effectively makes up for the current shortcomings of using a single classifier. Therefore, this study comprehensively and completely demonstrated the process of predicting the outcome of T2DM drug treatment with the help of data mining technology under the background of real-world research and provided a good methodological reference for the management of other chronic diseases.

### Strengths

1. The data in this study came from the Public Health Service System and the Medical Record Homepage Management System of the Health Information Center of Sichuan Province. The data quality is reliable enough to meet the needs of modeling.
2. In the process of data cleaning, this study is not limited to a single data preprocessing method but uses a variety of inputting methods, feature screening methods, and various data preprocessing methods and applies them to the data cleaning of each predictive indicator. The process avoids the possible impact of a certain data preprocessing method on the modeling effect.
3. The modeling method has been improved in this study. Different from the previous use of one or several algorithms to build predictive models, our study used more than ten machine learning algorithms for modeling and selected the optimal five models. The model results are more reliable and the prediction performance is better.
4. The information included in the study is more comprehensive, including the basic information about patients, disease-related factors, treatment factors, metabolic index factors, and lifestyle-related factors.

### Limitations

1. This study only uses medical data from Sichuan Province, China for modeling. Differences in lifestyle and ethnicity in different regions may lead to a limited scope of application of the model.
2. Although the data set used for validation in this study is independent of the data set used for model development, the two are derived from practice records in the same database, and no more rigorous prospective external validation has been performed.
3. In this study, the classification of some variables may be wrong because the system automatically recognizes variables with more than 10 categories as continuous variables, but this operation will not affect the prediction effect of the final prediction model.
4. In this study, the AUC values of the two optimal prediction models differed by 0.1, possibly due to too little HbA1c data for modeling. In future studies, if the amount of data used to build the model can be increased, the difference in values may be reduced.
5. Some predictive factors, such as the course of diabetes, are not recorded in detail in the original database, so these variables are not included in the modeling, which may have an impact on the prediction results. In the following research, we will use more extensive and detailed data for modeling to obtain a more accurate prediction model.

## Conclusion

In this study, the three-month FBG prediction model and three-month HbA1c prediction model for T2DM patients were constructed using population data from Sichuan Province, China. The patient's FBG and HbA1c are both the most important predictors of two kinds of prediction models. This research can provide a methodological reference for other prediction models. The AUC values of the five best FBG prediction models finally established are all greater than 0.8, and the AUC values of the five best HbA1c prediction models finally established are all greater than 0.9, which could accurately predict the FBG and HbA1c for clinical applications.

In our future plans, we intend to establish a web-based prediction platform that will integrate the best prediction models. This platform will allow the input of various factors strongly associated with glycemic control in T2DM, such as individual characteristics, disease status, medication information, and laboratory test results. Using the prediction platform, the prediction model can be applied in clinical practice for forecasting FBG and HbA1c control in patients, and our institution conducts preliminary application studies. Actually, it's challenging to ensure the absolute correctness of model predictions. Therefore, in clinical practice, the results of predictive models can be combined with the empirical knowledge of clinicians and pharmacists to help them make decisions to improve patient outcomes.

### Supplementary Information

Supplementary Information.

## Data Availability

The data of the Public Health Service System and the Medical Record Homepage Management System of the Health Information Center of Sichuan Province, China used to support the findings of this study have not been made available because the availability of these data is only licensed under the current study and therefore not publicly available. Data are however available from the authors upon reasonable request and with permission of Sichuan Provincial Health Commission.

## Abbreviations

ALT: Alanine aminotransferase;
AST: Aspartate aminotransferase
AUC: Area under the curve
BMI: Body mass index
BP-ANN: Back-propagation artificial neural network
BUN: Blood urea nitrogen
CI: Confidence intervals
CPM: Cycles per minute
DBP: Diastolic blood pressure
EN: Elastic network
FBG: Fasting blood glucose
Fig: Figure
Hb: Hemoglobin
HbA1c: Glycosylated hemoglobin
HBP: High blood pressure
HDL-C: High density lipoprotein cholesterol
LDA: Linear discriminant analysis
P-R curve: Precision-recall curve
QDA: Quadratic discriminant analysis
RF: Random forest
ROC: Receiver operating characteristic curve
SBP: Systolic blood pressure
Scr: Serum creatinine
SD: Standard deviation
SGD: Stochastic Gradient Descent
SVM: Support vector machine
TC: Total cholesterol
TRIPOD: Transparent reporting of a multivariable prediction model for individual prognosis or diagnosis
TG: Triglyceride
TP: Total protein
UA: Uric acid
