# Supplementary material for: Predicting three-month fasting blood glucose and glycated hemoglobin changes in patients with type 2 diabetes mellitus based on multiple machine learning algorithms
Source: Sci Rep. 2023 Sep 30;13:16437. doi: 10.1038/s41598-023-43240-5 (PMC10543442; doi:10.1038/s41598-023-43240-5)
Supplement: Supplementary file 1 — Supplementary Information. [file 41598_2023_43240_MOESM1_ESM.docx]

Supplementary material

**Table S1. Relevant variables and subgroup for the construction of the prediction model**

| Number | Variable | Unit | Subgroup |
| --- | --- | --- | --- |
| X1 | Age | years | - |
| X2 | Temperature | Celsius | - |
| X3 | Pulse rate | counts per minute | - |
| X4 | Respiratory frequency | counts per minute | - |
| X5 | Waist circumference | cm | - |
| X6 | BMI | kg/m^2^ | - |
| X7 | Self-assessment of self-care ability of the elderly | - | - |
| X8 | Cognitive function in the elderly | - | - |
| X9 | Mini-mental state examination | - | - |
| X10 | Depression | - | - |
| X11 | Elderly depression score examination | - | - |
| X12 | Time of each exercise | minutes | - |
| X13 | Exercise duration | minutes | - |
| X14 | Exercise mode | - | - |
| X15 | Daily smoking | cigarettes | - |
| X16 | Age of smoking initiation | years | - |
| X17 | Smoking cessation age | years | - |
| X18 | Daily alcohol intake | tael | - |
| X19 | Quit drink | - | - |
| X20 | Drunkenness in the past year | - | - |
| X21 | Left eye correction | - | - |
| X22 | Right eye correction | - | - |
| X23 | Hearing | - | - |
| X24 | Motor function | - | - |
| X25 | Description of fundus abnormalities | - | - |
| X26 | Hemoglobin | g/L | - |
| X27 | Leucocyte | ×10^9^/L | - |
| X28 | [Platelets](https://www.ncbi.nlm.nih.gov/mesh/68001792) | ×10^9^/L | - |
| X29 | Urine protein | mg/dL | - |
| X30 | Urine glucose | mg/dL | - |
| X31 | Ketone | mg/dL | - |
| X32 | Urinary occult blood | - | - |
| X33 | Urinary microalbumin | mg/L | - |
| X34 | Hemoglobin A1c(HbA1c) | % | - |
| X35 | HBsAg | ng/mL | - |
| X36 | Alanine aminotransferase(ALT) | U/L | - |
| X37 | Aspartate aminotransferase(AST) | U/L | - |
| X38 | Albumin | g/L | - |
| X39 | Total bilirubin | μmol/L | - |
| X40 | Conjugated bilirubin | μmol/L | - |
| X41 | Serum creatinine(Scr) | μmol/L | - |
| X42 | Blood urea nitrogen | mmol/L | - |
| X43 | Potassium | mmol/L | - |
| X44 | Sodium | mmol/L | - |
| X45 | Total cholesterol(TC) | mmol/L | - |
| X46 | Triglyceride(TG) | mmol/L | - |
| X47 | Low density lipoprotein cholesterol(LDL-C) | mmol/L | - |
| X48 | High density lipoprotein cholesterol(HDL-C) | mmol/L | - |
| X49 | Nervous system disease | - | - |
| X50 | Other systemic diseases | - | - |
| X51 | Symptoms | - | Dizziness and headache |
| X52 | Symptoms | - | Nausea and vomiting |
| X53 | Symptoms | - | Vertigo and tinnitus |
| X54 | Symptoms | - | Dyspnea |
| X55 | Symptoms | - | Palpitation and chest distress |
| X56 | Symptoms | - | Chest pain |
| X57 | Symptoms | - | Numbness of limbs |
| X58 | Symptoms | - | Lower limb oedema |
| X59 | Symptoms | - | Chronic cough |
| X60 | Symptoms | - | [Expectoration](http://www.letpub.com.cn/index.php?page=internal-medicine&med_id=4534&class_id=1) |
| X61 | Symptoms | - | Polydipsia |
| X62 | Symptoms | - | Polyuria |
| X63 | Symptoms | - | Weight loss |
| X64 | Symptoms | - | Malaise |
| X65 | Symptoms | - | Blurred vision |
| X66 | Symptoms | - | Urgent micturition |
| X67 | Symptoms | - | Dysuria |
| X68 | Symptoms | - | Constipation |
| X69 | Symptoms | - | Diarrhea |
| X70 | Symptoms | - | Breast distending pain |
| X71 | Symptoms | - | Other |
| X72 | Self-assessment of the health status of the elderly | - | Satisfaction |
| X73 | Self-assessment of the health status of the elderly | - | Basic satisfaction |
| X74 | Self-assessment of the health status of the elderly | - | Not clear |
| X75 | Self-assessment of the health status of the elderly | - | Not very satisfaction |
| X76 | Self-assessment of the health status of the elderly | - | Not satisfaction |
| X77 | Exercise frequency | - | Daily |
| X78 | Exercise frequency | - | More than once a week |
| X79 | Exercise frequency | - | Occasional |
| X80 | Exercise frequency | - | No exercise |
| X81 | Eating habits | - | Balanced meat and vegetables |
| X82 | Eating habits | - | Meat-based |
| X83 | Eating habits | - | Vegetable-based |
| X84 | Eating habits | - | Salt addiction |
| X85 | Eating habits | - | Oil addiction |
| X86 | Eating habits | - | Sugar addiction |
| X87 | Smoking status | - | Never |
| X88 | Smoking status | - | Smoking Cessation |
| X89 | Smoking status | - | Smoking |
| X90 | Drinking frequency | - | Never |
| X91 | Drinking frequency | - | Occasional |
| X92 | Drinking frequency | - | Frequent |
| X93 | Drinking frequency | - | Daily |
| X94 | Type of drinking | - | Liquor |
| X95 | Type of drinking | - | Beer |
| X96 | Type of drinking | - | Red wine |
| X97 | Type of drinking | - | Rice wine |
| X98 | Type of drinking | - | Other |
| X99 | Cardiac rhythm | - | Regular |
| X100 | Cardiac rhythm | - | Arrhythmia |
| X101 | Cardiac rhythm | - | Absolute arrhythmia |
| X102 | Lower limb oedema | - | Unilateral |
| X103 | Lower limb oedema | - | Bilateral asymmetry |
| X104 | Lower limb oedema |  | Bilateral symmetry |
| X105 | Dorsalis pedis pulse | - | Not palpable |
| X106 | Dorsalis pedis pulse | - | Palpation symmetry |
| X107 | Dorsalis pedis pulse | - | Weak or absent to left |
| X108 | Dorsalis pedis pulse | - | Weak or absent to right |
| X109 | Mammary gland | - | No abnormalities |
| X110 | Mammary gland | - | Mastectomy |
| X111 | Mammary gland | - | Abnormal lactation |
| X112 | Mammary gland | - | Masses |
| X113 | Mammary gland | - | Other |
| X114 | Cerebrovascular disease | - | Not found |
| X115 | Cerebrovascular disease | - | Ischemic stroke |
| X116 | Cerebrovascular disease | - | Cerebral hemorrhage |
| X117 | Cerebrovascular disease | - | Subarachnoid hemorrhage |
| X118 | Cerebrovascular disease | - | Transient ischemic attack |
| X119 | Cerebrovascular disease | - | Other |
| X120 | Kidney disease | - | Not found |
| X121 | Kidney disease | - | Diabetic Nephropathy(DN) |
| X122 | Kidney disease | - | Renal failure |
| X123 | Kidney disease | - | Acute nephritis |
| X124 | Kidney disease | - | Chronic nephritis |
| X125 | Kidney disease | - | Other |
| X126 | Heart disease | - | Not found |
| X127 | Heart disease | - | Myocardial infarction |
| X128 | Heart disease | - | Angina pectoris |
| X129 | Heart disease | - | Coronary revascularization |
| X130 | Heart disease | - | Congestive heart failure |
| X131 | Heart disease | - | Precordial pain |
| X132 | Heart disease | - | Other |
| X133 | Vascular disease | - | Not found |
| X134 | Vascular disease | - | Dissecting aneurysm |
| X135 | Vascular disease | - | Arterial occlusive disease |
| X136 | Vascular disease | - | Other |
| X137 | Eye disease | - | Not found |
| X138 | Eye disease | - | Retinal hemorrhage or exudate |
| X139 | Eye disease | - | Papilledema |
| X140 | Eye disease | - | Cataract |
| X141 | Eye disease | - | Other |
| X142 | Risk factor control | - | Smoking cessation |
| X143 | Risk factor control | - | Healthy drinking |
| X144 | Risk factor control | - | Diet |
| X145 | Risk factor control | - | Exercise |
| X146 | Risk factor control | - | Weight loss goal |
| X147 | Risk factor control | - | Vaccination |
| X148 | Risk factor control | - | Other |
| X149 | Other | - | Other abnorma |
| X150 | Fasting blood glucose(FBG) | mmol/L | - |
| X151 | Blood pressure | mmHg | Diastolic blood pressure(DBP) |
| X152 | Blood pressure | mmHg | Systolic blood pressure(SBP) |
| X153 | Current daily smoking | cigarettes | Average daily |
| X154 | Current daily alcohol intake | tael | Average daily |
| X155 | Current time of each exercise | minutes | Average daily exercise |
| X156 | Current number of exercise per week | times | - |
| X157 | Current staple food | g/day | Average daily |
| X158 | Adverse drug reaction | - | - |
| X159 | Symptoms | - | Epistaxis continuous bleeding |
| X160 | Follow-up method | - | Outpatient |
| X161 | Follow-up method | - | Home visit |
| X162 | Follow-up method | - | Telephone |
| X163 | Dorsalis pedis pulse | - | Normal |
| X164 | Dorsalis pedis pulse | - | Weak |
| X165 | Dorsalis pedis pulse | - | Disappearance |
| X166 | Mental adjustment | - | Good |
| X167 | Mental adjustment | - | General |
| X168 | Mental adjustment | - | Poor |
| X169 | Medical compliance | - | Good |
| X170 | Medical compliance | - | General |
| X171 | Medical compliance | - | Poor |
| X172 | Medication adherence | - | Regularity |
| X173 | Medication adherence | - | Intermittent |
| X174 | Medication adherence | - | No medication |
| X175 | Hypoglycemic reaction | - | - |
| X176 | Hypoglycemic reaction | - | Occasional |
| X177 | Hypoglycemic reaction | - | Frequent |
| X178 | Classification of this follow-up | - | Satisfaction |
| X179 | Classification of this follow-up | - | Adverse reactions |
| X180 | Classification of this follow-up | - | Complication |
| X181 | Sex | - | - |
| X182 | national | - | - |
| X183 | Education | - | - |
| X184 | Profession | - | - |
| X185 | Marital status | - | - |
| X186 | Surgery | - | - |
| X187 | Trauma | - | - |
| X188 | History of genetic disease | - | - |
| X189 | ABO blood group | - | Blood type A |
| X190 | ABO blood group | - | Blood type B |
| X191 | ABO blood group | - | Blood type O |
| X192 | ABO blood group | - | Blood type AB |
| X193 | Rh blood group | - | Not clear |
| X194 | Rh blood group | - | Negative |
| X195 | Rh blood group | - | Positive |
| X196 | History of drug allergy | - | Penicillin |
| X197 | History of drug allergy | - | Sulphonamide |
| X198 | History of drug allergy | - | Streptomycin |
| X199 | History of drug allergy | - | Other |
| X200 | History of exposure | - | chemical |
| X201 | History of exposure | - | toxicant |
| X202 | History of exposure | - | ray |
| X203 | History of exposure | - | Other |
| X204 | Disease | - | Hypertension |
| X205 | Disease | - | Diabetes Mellitus(DM) |
| X206 | Disease | - | Coronary heart disease |
| X207 | Disease | - | Chronic obstructive pulmonary disease |
| X208 | Disease | - | Malignant tumour |
| X209 | Disease | - | Stroke |
| X210 | Disease | - | Severe mental disorder |
| X211 | Disease | - | Tuberculosis |
| X212 | Disease | - | Hepatitis |
| X213 | Disease | - | Other notifiable infectious diseases |
| X214 | Disease | - | Occupation disease |
| X215 | Disease | - | Other |
| X216 | Father's disease | - | Hypertension |
| X217 | Father's disease | - | Diabetes Mellitus(DM) |
| X218 | Father's disease | - | Coronary heart disease |
| X219 | Father's disease | - | Chronic obstructive pulmonary disease |
| X220 | Father's disease | - | Malignant tumour |
| X221 | Father's disease | - | Stroke |
| X222 | Father's disease | - | Severe mental disorder |
| X223 | Father's disease | - | Tuberculosis |
| X224 | Father's disease | - | Hepatitis |
| X225 | Father's disease | - | Congenital abnormalities |
| X226 | Father's disease | - | Other |
| X227 | Maternal disease | - | Hypertension |
| X228 | Maternal disease | - | Diabetes Mellitus(DM) |
| X229 | Maternal disease | - | Coronary heart disease |
| X230 | Maternal disease | - | Chronic obstructive pulmonary disease |
| X231 | Maternal disease | - | Malignant tumour |
| X232 | Maternal disease | - | Stroke |
| X233 | Maternal disease | - | Severe mental disorder |
| X234 | Maternal disease | - | Tuberculosis |
| X235 | Maternal disease | - | Hepatitis |
| X236 | Maternal disease | - | Congenital abnormalities |
| X237 | Maternal disease | - | Other |
| X238 | Sibling disease | - | Hypertension |
| X239 | Sibling disease | - | Diabetes Mellitus(DM) |
| X240 | Sibling disease | - | Coronary heart disease |
| X241 | Sibling disease | - | Chronic obstructive pulmonary disease |
| X242 | Sibling disease | - | Malignant tumour |
| X243 | Sibling disease | - | Stroke |
| X244 | Sibling disease | - | Severe mental disorder |
| X245 | Sibling disease | - | Tuberculosis |
| X246 | Sibling disease | - | Hepatitis |
| X247 | Sibling disease | - | Congenital abnormalities |
| X248 | Sibling disease | - | Other |
| X249 | Disease of child | - | Hypertension |
| X250 | Disease of child | - | Diabetes Mellitus(DM) |
| X251 | Disease of child | - | Coronary heart disease |
| X252 | Disease of child | - | Chronic obstructive pulmonary disease |
| X253 | Disease of child | - | Malignant tumour |
| X254 | Disease of child | - | Stroke |
| X255 | Disease of child | - | Severe mental disorder |
| X256 | Disease of child | - | Tuberculosis |
| X257 | Disease of child | - | Hepatitis |
| X258 | Disease of child | - | Congenital abnormalities |
| X259 | Disease of child | - | Other |
| X260 | Disability | - | visual disability |
| X261 | Disability | - | Hearing disability |
| X262 | Disability | - | Speech disability |
| X263 | Disability | - | Physical disability |
| X264 | Disability | - | Intellectual disability |
| X265 | Disability | - | Mental disability |
| X266 | Disability | - | Other |
| X267 | Atherosclerotic cardiovascular disease | - | - |
| X268 | Metformin | - | - |
| X269 | Metformin sustained-release capsules | - | - |
| X270 | Metformin sustained-release tablets | - | - |
| X271 | Phenformin | - | - |
| X272 | Tolbutamide | - | - |
| X273 | Glibenclamide | - | - |
| X274 | Glibenclamide sustained-release tablets | - | - |
| X275 | Metformin and glibenclamide | - | - |
| X276 | Metformin and glibenclamide sustained-release | - | - |
| X277 | Glimepirde | - | - |
| X278 | Glimepirde sustained-release | - | - |
| X279 | Metformin and Glimepirde | - | - |
| X280 | Metformin and Glimepirde sustained-release | - | - |
| X281 | Metformin and glipizide | - | - |
| X282 | Gliclazide sustained-release | - | - |
| X283 | Metformin and Gliclazide | - | - |
| X284 | Metformin and glipizide sustained-release tablets | - | - |
| X285 | Gliclazide tablets | - | - |
| X286 | Glipizide tablets | - | - |
| X287 | Glipizide controlled release tablets | - | - |
| X288 | Gliquidone tablets | - | - |
| X289 | Metformin and gliquidone dispersible tablets | - | - |
| X290 | Pioglitazone tablets | - | - |
| X291 | Pioglitazone tablets 15mg | - | - |
| X292 | Pioglitazone tablets 30mg | - | - |
| X293 | Pioglitazone sustained-release tablets | - | - |
| X294 | Pioglitazone and metformin tablets (15mg/500mg) | - | - |
| X295 | Pioglitazone and metformin tablets | - | - |
| X296 | Pioglitazone and metformin sustained-release tablets (15mg/500mg) | - | - |
| X297 | Pioglitazone and metformin sustained-release tablets | - | - |
| X298 | Rosiglitazone tablets | - | - |
| X299 | Rosiglitazone sustained-release tablets | - | - |
| X300 | Metformin and rosiglitazone tablets | - | - |
| X301 | Metformin hydrochloride and rosiglitazone maleate sustained-release tablets | - | - |
| X302 | Repaglinide tablets | - | - |
| X303 | GLP-1 receptor agonist | - | - |
| X304 | Metformin and vildagliptin | - | - |
| X305 | Vildagliptin tablets 50mg | - | - |
| X306 | Vildagliptin tablets | - | - |
| X307 | Sitagliptin | - | - |
| X308 | Sitagliptin and metformin | - | - |
| X309 | Empagliflozin | - | - |
| X310 | Mecobalamin | - | - |
| X311 | Epalrestat | - | - |
| X312 | Miglitol | - | - |
| X313 | Voglibose | - | - |
| X314 | Acarbose | - | - |
| X315 | Insulin aspart | - | - |
| X316 | Insulin lispro | - | - |
| X317 | Insulin glulisine | - | - |
| X318 | NovoRapid | - | - |
| X319 | Other rapid-acting insulins | - | - |
| X320 | Short-acting insulin | - | - |
| X321 | Protamine Zinc Insulin | - | - |
| X322 | Insulin glargine | - | - |
| X323 | Regular recombinant human insulin | - | - |
| X324 | Insulin detemir | - | - |
| X325 | Isophane recombinant human insulin | - | - |
| X326 | Long-acting insulin | - | - |
| X327 | Recombinant human insulin injection (mixed 30/70) | - | - |
| X328 | Pre-Mixed insulin | - | - |
| X329 | Other types of insulin | - | - |
| X330 | Chinese patent drug | - | - |
| X331 | Other types antidiabetic drugs | - | - |
| X332 | Levamlodipine | - | - |
| X333 | Nifedipine | - | - |
| X334 | Nifedipine tablets | - | - |
| X335 | Nifedipine sustained-release tablets | - | - |
| X336 | Lacidipine | - | - |
| X337 | Felodipine | - | - |
| X338 | Felodipine sustained-release | - | - |
| X339 | Nimodipine tablets | - | - |
| X340 | Nimodipine | - | - |
| X341 | Nitrendipine | - | - |
| X342 | Amlodipine and Benazepril | - | - |
| X343 | Benazepril | - | - |
| X344 | Fosinopril | - | - |
| X345 | Captopril | - | - |
| X346 | Perindopril | - | - |
| X347 | Lisinopril | - | - |
| X348 | Bisoprolol | - | - |
| X349 | Bisoprolol (Dosage and administration are not distinguished) | - | - |
| X350 | Metoprolol | - | - |
| X351 | Propranolol tablets | - | - |
| X352 | Propranolol capsules | - | - |
| X353 | Candesartan | - | - |
| X354 | Valsartan | - | - |
| X355 | Telmisartan tablets 80mg | - | - |
| X356 | Telmisartan | - | - |
| X357 | Irbesartan | - | - |
| X358 | Irbesartan tablets | - | - |
| X359 | Irbesartan and hydrochlorothiazide | - | - |
| X360 | Irbesartan tablets and hydrochlorothiazide | - | - |
| X361 | Losartan potassium | - | - |
| X362 | Losartan | - | - |
| X363 | Compound reserpine tablets | - | - |
| X364 | Reserpine tablets | - | - |
| X365 | Reserpine | - | - |
| X366 | Hydrochlorothiazide tablets | - | - |
| X367 | Hydrochlorothiazide | - | - |
| X368 | Spironolactone tablets | - | - |
| X369 | Spironolactone | - | - |
| X370 | Indapamide | - | - |
| X371 | Indapamide tablets | - | - |
| X372 | Indapamide sustained-release | - | - |
| X373 | Compound reserpineand triamterene tablets | - | - |
| X374 | Triamterene tablets | - | - |
| X375 | Triamterene | - | - |
| X376 | Antihypertensive Chinese patent medicines | - | - |
| X377 | Compound Danshen pills | - | - |
| X378 | Compound Danshen tablets | - | - |
| X379 | Compound Danshen | - | - |
| X380 | Ginkgo folium | - | - |
| X381 | Atorvastatin calcium | - | - |
| X382 | Atorvastatin | - | - |
| X383 | Rosuvastatin | - | - |
| X384 | Simvastatin tablets | - | - |
| X385 | Simvastatin tablets 20mg | - | - |
| X386 | Pravastatin | - | - |
| X387 | Pitavastatin | - | - |
| X388 | Other statins | - | - |
| X389 | Fenofibrate | - | - |
| X390 | Aspirin | - | - |
| X391 | Clopidogrel | - | - |
| X392 | Cardiovascular proprietary Chinese medicine | - | - |
| X393 | Propolis nourishes trypsin | - | - |
| X394 | Propolis capsules | - | - |
| X395 | Propolis | - | - |
| X396 | Nitroglycerin tablets | - | - |
| X397 | Isosorbide dinitrate tablets | - | - |
| X398 | Isosorbide mononitrate tablets | - | - |
| X399 | Isosorbide mononitrate sustained-release tablets | - | - |
| X400 | Nitrates | - | - |
| X401 | Lumbrokinase enteric - coated capsules | - | - |
| X402 | Calcium dobesilate capsules | - | - |
| X403 | Type 2 diabetes mellitus | - | - |
| X404 | Other diabetes types (not used) | - | - |
| X405 | Diabetes distal symmetrical polyneuropathy | - | - |
| X406 | Ophthalmopathy | - | - |
| X407 | Diabetic nephropathy | - | - |
| X408 | Diabetic foot | - | - |
| X409 | Diabetic ketoacidosis | - | - |
| X410 | Hypoglycemia | - | - |
| X411 | T2DM with other complications | - | - |
| X412 | Other diabetes types (Delete this part of patients) | - | - |
| X413 | Hypertension | - | - |
| X414 | Atherosclerosis | - | - |
| X415 | Hyperlipidemias | - | - |
| X416 | Coronary atherosclerotic heart disease | - | - |
| X417 | Heart failure | - | - |
| X418 | Angina pectoris | - | - |
| X419 | Myocardial infarction | - | - |
| X420 | Arrhythmia | - | - |
| X421 | Heart valve diseases | - | - |
| X422 | Cardiomyopathies | - | - |
| X423 | Pericardial disease | - | - |
| X424 | Hypertensive heart disease | - | - |
| X425 | Congenital cardiovascular disease | - | - |
| X426 | Rheumatic heart disease | - | - |
| X427 | Pulmonary heart disease | - | - |
| X428 | Myocardial ischemia | - | - |
| X429 | Other diseases of circulatory system | - | - |
| X430 | Stroke | - | - |
| X431 | Cerebral infarction | - | - |
| X432 | Cerebral hemorrhage | - | - |
| X433 | Transient ischemic attack | - | - |
| X434 | Cerebral atherosclerosis | - | - |
| X435 | Subarachnoid hemorrhage | - | - |
| X436 | Other cerebrovascular diseases | - | - |
| X437 | Epilepsy | - | - |
| X438 | Parkinsonism | - | - |
| X439 | Alzheimer's disease | - | - |
| X440 | Brain neoplasms | - | - |
| X441 | Other encephalopathy | - | - |
| X442 | Chronic obstructive pulmonary disease | - | - |
| X443 | acute tracheitis or acute tracheitis | - | - |
| X444 | Chronic bronchitis | - | - |
| X445 | Pulmonary emphysema | - | - |
| X446 | Bronchiectasis | - | - |
| X447 | Asthma | - | - |
| X448 | Disease of pleura | - | - |
| X449 | Pneumonia | - | - |
| X450 | Pneumoconiosis | - | - |
| X451 | Silicosis | - | - |
| X452 | Lung neoplasms | - | - |
| X453 | Tuberculosis | - | - |
| X454 | Rhinitis | - | - |
| X455 | Other diseases of respiratory system | - | - |
| X456 | Nephritis | - | - |
| X457 | Renal failure | - | - |
| X458 | Renal cyst | - | - |
| X459 | Nephrolithiasis | - | - |
| X460 | Other kidney diseases | - | - |
| X461 | Ureteral calculi | - | - |
| X462 | Uremia | - | - |
| X463 | Prostatitis | - | - |
| X464 | Prostatic hyperplasia | - | - |
| X465 | Other prostate diseases | - | - |
| X466 | Bladder disease | - | - |
| X467 | Anemia | - | - |
| X468 | Lymphoma | - | - |
| X469 | Other blood system diseases | - | - |
| X470 | Metabolic liver disease | - | - |
| X471 | Hepatitis | - | - |
| X472 | Hepatic space-occupying lesions | - | - |
| X473 | Hepatic cyst | - | - |
| X474 | Liver Neoplasms | - | - |
| X475 | Liver cirrhosis | - | - |
| X476 | Hepatic calcification | - | - |
| X477 | Hepatic insufficiency | - | - |
| X478 | Hepatic congestion | - | - |
| X479 | Other diseases of liver | - | - |
| X480 | Gallstones | - | - |
| X481 | Absence of gallbladder | - | - |
| X482 | Cholecystectomy | - | - |
| X483 | Gallbladder polyp | - | - |
| X484 | Cholangiectasis | - | - |
| X485 | Other biliary diseases | - | - |
| X486 | Gastritis | - | - |
| X487 | Peptic ulcer | - | - |
| X488 | Gastrointestinal haemorrhage | - | - |
| X489 | Pancreatitis | - | - |
| X490 | Other diseases of pancreas | - | - |
| X491 | Other diseases of digestive system | - | - |
| X492 | Hypothyroidism | - | - |
| X493 | Hyperthyroidism | - | - |
| X494 | Other thyroid diseases | - | - |
| X495 | Hyperuricemia | - | - |
| X496 | Gout | - | - |
| X497 | Rheumatic arthritides | - | - |
| X498 | Rheumatoid arthritis | - | - |
| X499 | Osteoarthritis | - | - |
| X500 | Scapulohumeral periarthritis | - | - |
| X501 | Synovitis | - | - |
| X502 | Spondylosis | - | - |
| X503 | Lumbar spondylosis | - | - |
| X504 | Fracture | - | - |
| X505 | Other diseases in orthopedics | - | - |
| X506 | Hyperostosis | - | - |
| X507 | Osteoporosis | - | - |
| X508 | Uterine leiomyoma | - | - |
| X509 | Other diseases of the uterus | - | - |
| X510 | Cataract | - | - |
| X511 | Eczema | - | - |

**Table S2 Delete variables**

| FBG | | | HbA1c | | |
| --- | --- | --- | --- | --- | --- |
| Missing ratio >90%（N=12） | single category ratio＞ 90%（N=412） | coefficient of variatio＜0.1（N=2） | Missing ratio >90%（N=20） | single category ratio＞ 90%（N=410） | coefficient of variatio＜0.1（N=2） |
| X9，X11，X17，X21，X22，X29，X31，X32，X33，X43，X44，X49 | X24，X53，X54，X55，X56，X57，X58，X59，X60，X61，X62，X63，X64，X65，X66，X67，X68，X69，X70，X71，X74，X75，X76，X78，X79，X81，X83，X84，X85，X86，X88，X91，X92，X93，X95，X96，X97，X98，X100，X101，X102，X103，X104，X105，X107，X108，X109，X110，X111，X112，X113，X115，X116，X117，X118，X119，X121，X122，X123，X124，X125，X127，X128，X129，X130，X131，X132，X134，X135，X136，X138，X139，X140，X141，X142，X143，X145，X146，X147，X148，X149，X158，X159，X162，X163，X164，X165，X168，X171，X175，X176，X177，X180，X182，X188，X189，X190，X191，X192，X193，X194，X195，X196，X197，X198，X199，X200，X201，X202，X203，X205，X206，X207，X208，X209，X210，X211，X212，X213，X214，X216，X217，X218，X219，X220，X221，X222，X223，X224，X225，X226，X227，X228，X229，X230，X231，X232，X233，X234，X235，X236，X237，X238，X239，X240，X241，X242，X243，X244，X245，X246，X247，X248，X249，X250，X251，X252，X253，X254，X255，X256，X257，X258，X259，X260，X261，X262，X263，X264，X265，X266，X267，X270，X271，X272，X273，X274，X275，X276，X277，X278，X279，X280，X281，X282，X283，X284，X286，X287，X288，X289，X290，X291，X292，X293，X294，X295，X296，X297，X298，X299，X300，X301，X302，X303，X304，X305，X306，X307，X308，X309，X310，X311，X312，X313，X314，X315，X316，X317，X318，X319，X320，X321，X322，X323，X324，X325，X326，X327，X328，X329，X330，X331，X332，X333，X334，X335，X336，X337，X338，X339，X340，X341，X342，X343，X344，X345，X346，X347，X348，X349，X350，X351，X352，X353，X354，X355，X356，X357，X358，X359，X360，X361，X362，X363，X364，X365，X366，X367，X368，X369，X370，X371，X372，X373，X374，X375，X376，X377，X378，X379，X380，X381，X382，X383，X384，X385，X386，X387，X388，X389，X390，X391，X392，X393，X394，X395，X396，X397，X398，X399，X400，X401，X402，X404，X405，X406，X407，X408，X409，X410，X411，X412，X413，X414，X415，X416，X417，X418，X419，X420，X421，X422，X423，X424，X425，X426，X427，X428，X429，X430，X431，X432，X433，X434，X435，X436，X437，X438，X439，X440，X441，X442，X443，X444，X445，X446，X447，X448，X449，X450，X451，X452，X453，X454，X455，X456，X457，X458，X459，X460，X461，X462，X463，X464，X465，X466，X467，X468，X469，X470，X471，X472，X473，X474，X475，X476，X477，X478，X479，X480，X481，X482，X483，X484，X485，X486，X487，X488，X489，X490，X491，X492，X493，X494，X495，X496，X497，X498，X499，X500，X501，X502，X503，X504，X505，X506，X507，X508，X509，X510，X511 | X2，X4 | X9，X11，X15，X16，X17，X18，X19，X21，X22，X29，X30，X31，X32，X33，X35，X38，X40，X43，X44，X49 | X53，X54，X55，X56，X57，X58，X59，X60，X61，X62，X63，X64，X65，X66，X67，X68，X69，X70，X71，X73，X74，X75，X76，X78，X79，X81，X83，X84，X85，X86，X88，X89，X91，X92，X93，X94，X95，X96，X97，X98，X100，X101，X102，X103，X104，X105，X107，X108，X109，X110，X111，X112，X113，X115，X116，X117，X118，X119，X121，X122，X123，X124，X127，X128，X129，X130，X131，X132，X134，X135，X136，X138，X139，X140，X141，X142，X143，X145，X146，X147，X148，X158，X159，X162，X163，X164，X165，X168，X175，X176，X177，X180，X182，X188，X189，X190，X191，X192，X193，X194，X195，X196，X197，X198，X199，X200，X201，X202，X203，X205，X206，X207，X208，X209，X210，X211，X212，X213，X214，X216，X217，X218，X219，X220，X221，X222，X223，X224，X225，X226，X227，X228，X229，X230，X231，X232，X233，X234，X235，X236，X237，X238，X240，X241，X242，X243，X244，X245，X246，X247，X248，X249，X250，X251，X252，X253，X254，X255，X256，X257，X258，X259，X260，X261，X262，X263，X264，X265，X266，X267，X268，X269，X270，X271，X272，X273，X274，X275，X276，X277，X278，X279，X280，X281，X282，X283，X284，X285，X286，X287，X288，X289，X290，X291，X292，X293，X294，X295，X296，X297，X298，X299，X300，X301，X302，X303，X304，X305，X306，X307，X308，X309，X310，X311，X312，X313，X314，X315，X316，X317，X318，X319，X320，X321，X322，X323，X324，X325，X326，X327，X328，X329，X330，X331，X332，X333，X334，X335，X336，X337，X338，X339，X340，X341，X342，X343，X344，X345，X346，X347，X348，X349，X350，X351，X352，X353，X354，X355，X356，X357，X358，X359，X360，X361，X362，X363，X364，X365，X366，X367，X368，X369，X370，X371，X372，X373，X374，X375，X376，X377，X378，X379，X380，X381，X382，X383，X384，X385，X386，X387，X388，X389，X390，X391，X392，X393，X394，X395，X396，X397，X398，X399，X400，X401，X402，X404，X405，X406，X407，X408，X409，X410，X411，X412，X413，X414，X415，X416，X417，X418，X419，X420，X421，X422，X423，X424，X425，X426，X427，X428，X429，X430，X431，X432，X433，X434，X435，X436，X437，X438，X439，X440，X441，X442，X443，X444，X445，X446，X447，X448，X449，X450，X451，X452，X453，X454，X455，X456，X457，X458，X460，X461，X462，X463，X464，X465，X466，X467，X468，X469，X471，X472，X473，X474，X475，X476，X477，X478，X479，X480，X481，X482，X483，X484，X485，X486，X487，X488，X489，X490，X491，X492，X493，X494，X495，X496，X497，X498，X499，X500，X501，X502，X503，X504，X505，X506，X507，X508，X509，X510 | X2，X4 |

**Table S3 Inclusion variables**

| Final inclusion variables | FBG(N=85) | X1，X3，X5，X6，X7，X8，X10，X12，X13，X14，X15，X16，X18，X19，X20，X23，X25，X26，X27，X28，X30，X34，X35，X36，X37，X38，X39，X40，X41，X42，X45，X46，X47，X48，X50，X51，X52，X72，X73，X77，X80，X82，X87，X89，X90，X94，X99，X106，X114，X120，X126，X133，X137，X144，X150，X151，X152，X153，X154，X155，X156，X157，X160，X161，X166，X167，X169，X170，X172，X173，X174，X178，X179，X181，X183，X184，X185，X186，X187，X204，X215，X268，X269，X285，X403 |
| --- | --- | --- |
|  | HbA1c(N=78) | X1，X3，X5，X6，X7，X8，X10，X12，X13，X14，X15，X16，X18，X19，X23，X25，X26，X27，X28，X34，X35，X36，X37，X38，X39，X40，X41，X42，X45，X46，X47，X48，X50，X51，X52，X72，X73，X77，X80，X82，X87，X89，X90，X94，X99，X106，X120，X126，X144，X150，X151，X152，X153，X154，X155，X158，X159，X164，X165，X167，X168，X170，X171，X172，X176，X177，X179，X181，X182，X183，X184，X202，X213，X266，X267，X283，X401，X510 |

**Table S4. Ten-fold cross-validation analysis results for the data imputing method**

| **FBG** | **AUC** | | **Accuracy** | | **Precision** | | **Recall** | | **F1Score** | |
| --- | --- | --- | --- | --- | --- | --- | --- | --- | --- | --- |
|  | **Mean±SD** | **95%CI** | **Mean±SD** | **95%CI** | **Mean±SD** | **95%CI** | **Mean±SD** | **95%CI** | **Mean±SD** | **95%CI** |
| **Modified Random Forest** | **0.749±0.044** | **0.745-0.753** | **0.688±0.035** | **0.684-0.691** | **0.693±0.049** | **0.689-0.698** | **0.683±0.042** | **0.679-0.687** | **0.686±0.031** | **0.684-0.689** |
| **Not** | **0.710±0.038** | **0.706-0.713** | **0.662±0.030** | **0.660-0.665** | **0.649±0.033** | **0.646-0.652** | **0.639±0.039** | **0.635-0.642** | **0.644±0.034** | **0.640-0.647** |
| **P value** | **P<0.0001** | | **P<0.0001** | | **P<0.0001** | | **P<0.0001** | | **P<0.0001** | |

| **HbA1c** | **AUC** | | **Accuracy** | | **Precision** | | **Recall** | | **F1Score** | |
| --- | --- | --- | --- | --- | --- | --- | --- | --- | --- | --- |
|  | **Mean±SD** | **95%CI** | **Mean±SD** | **95%CI** | **Mean±SD** | **95%CI** | **Mean±SD** | **95%CI** | **Mean±SD** | **95%CI** |
| **Modified Random Forest** | **0.901±0.078** | **0.894-0.907** | **0.844±0.099** | **0.836-0.853** | **0.834±0.103** | **0.825-0.842** | **0.848±0.112** | **0.838-0.857** | **0.838±0.102** | **0.829-0.847** |
| **Not** | **0.622±0.123** | **0.611-0.633** | **0.606±0.097** | **0.598-0.615** | **0.651±0.094** | **0.643-0.659** | **0.736±0.168** | **0.722-0.751** | **0.682±0.105** | **0.673-0.691** |
| **P value** | **P<0.0001** | | **P<0.0001** | | **P<0.0001** | | **P<0.0001** | | **P<0.0001** | |

**Table S5. Ten-fold cross-validation analysis results of the feature screening method**

| **FBG** | **AUC** | | **Accuracy** | | **Precision** | | **Recall** | | **F1Score** | |
| --- | --- | --- | --- | --- | --- | --- | --- | --- | --- | --- |
|  | **Mean±SD** | **95%CI** | **Mean±SD** | **95%CI** | **Mean±SD** | **95%CI** | **Mean±SD** | **95%CI** | **Mean±SD** | **95%CI** |
| **Boruta** | **0.725±0.044** | **0.721-0.728** | **0.672±0.034** | **0.669-0.675** | **0.667±0.041** | **0.663-0.670** | **0.675±0.050** | **0.671-0.680** | **0.670±0.039** | **0.667-0.673** |
| **Lasso** | **0.728±0.038** | **0.725-0.731** | **0.674±0.029** | **0.671-0.676** | **0.670±0.038** | **0.667-0.673** | **0.674±0.041** | **0.670-0.677** | **0.671±0.032** | **0.668-0.673** |
| **Not** | **0.724±0.043** | **0.721-0.727** | **0.671±0.033** | **0.668-0.673** | **0.666±0.041** | **0.663-0.669** | **0.673±0.038** | **0.670-0.676** | **0.669±0.033** | **0.666-0.672** |
| **P value** | **P=0.2424** | | **P=0.1056** | | **P=0.0950** | | **P=0.0136** | | **P=0.0677** | |

| **HbA1c** | **AUC** | | **Accuracy** | | **Precision** | | **Recall** | | **F1Score** | |
| --- | --- | --- | --- | --- | --- | --- | --- | --- | --- | --- |
|  | **Mean±SD** | **95%CI** | **Mean±SD** | **95%CI** | **Mean±SD** | **95%CI** | **Mean±SD** | **95%CI** | **Mean±SD** | **95%CI** |
| **Boruta** | **0.753±0.142** | **0.743-0.764** | **0.712±0.126** | **0.703-0.721** | **0.721±0.124** | **0.711-0.730** | **0.725±0.134** | **0.715-0.735** | **0.718±0.116** | **0.709-0.727** |
| **Lasso** | **0.776±0.130** | **0.766-0.785** | **0.719±0.126** | **0.709-0.728** | **0.720±0.130** | **0.711-0.730** | **0.750±0.154** | **0.738-0.761** | **0.728±0.124** | **0.718-0.737** |
| **Not** | **0.735±0.145** | **0.724-0.746** | **0.688±0.132** | **0.678-0.698** | **0.698±0.130** | **0.688-0.708** | **0.715±0.150** | **0.704-0.727** | **0.699±0.120** | **0.690-0.708** |
| **P value** | **P<0.0001** | | **P<0.0001** | | **P=0.0009** | | **P<0.0001** | | **P<0.0001** | |

**Table S6. Bootstrapping sampling analysis results of data imputing method**

| **FBG** | **AUC** | | **Accuracy** | | **Precision** | | **Recall** | | **F1Score** | |
| --- | --- | --- | --- | --- | --- | --- | --- | --- | --- | --- |
|  | **Mean±SD** | **95%CI** | **Mean±SD** | **95%CI** | **Mean±SD** | **95%CI** | **Mean±SD** | **95%CI** | **Mean±SD** | **95%CI** |
| **Modified Random Forest** | **0.754±0.048** | **0.754-0.755** | **0.693±0.038** | **0.693-0.694** | **0.701±0.052** | **0.701-0.701** | **0.683±0.037** | **0.683-0.683** | **0.691±0.033** | **0.690-0.691** |
| **Not** | **0.715±0.034** | **0.715-0.716** | **0.668±0.027** | **0.668-0.668** | **0.653±0.030** | **0.653-0.654** | **0.650±0.030** | **0.650-0.650** | **0.651±0.028** | **0.651-0.652** |
| **P value** | **P<0.0001** | | **P<0.0001** | | **P<0.0001** | | **P<0.0001** | | **P<0.0001** | |

| **HbA1c** | **AUC** | | **Accuracy** | | **Precision** | | **Recall** | | **F1Score** | |
| --- | --- | --- | --- | --- | --- | --- | --- | --- | --- | --- |
|  | **Mean±SD** | **95%CI** | **Mean±SD** | **95%CI** | **Mean±SD** | **95%CI** | **Mean±SD** | **95%CI** | **Mean±SD** | **95%CI** |
| **Modified Random Forest** | **0.902±0.083** | **0.902-0.903** | **0.849±0.099** | **0.848-0.849** | **0.822±0.100** | **0.822-0.823** | **0.876±0.109** | **0.875-0.876** | **0.845±0.100** | **0.845-0.846** |
| **Not** | **0.652±0.094** | **0.651-0.652** | **0.628±0.080** | **0.627-0.628** | **0.679±0.090** | **0.678-0.679** | **0.725±0.113** | **0.724-0.726** | **0.695±0.077** | **0.694-0.695** |
| **P value** | **P<0.0001** | | **P<0.0001** | | **P<0.0001** | | **P<0.0001** | | **P<0.0001** | |

**Table S7.Bootstrapping Sampling Analysis Results of Feature Screening Method**

| **FBG** | **AUC** | | **Accuracy** | | **Precision** | | **Recall** | | **F1Score** | |
| --- | --- | --- | --- | --- | --- | --- | --- | --- | --- | --- |
|  | **Mean±SD** | **95%CI** | **Mean±SD** | **95%CI** | **Mean±SD** | **95%CI** | **Mean±SD** | **95%CI** | **Mean±SD** | **95%CI** |
| **Boruta** | **0.732±0.040** | **0.731-0.732** | **0.679±0.031** | **0.678-0.679** | **0.674±0.041** | **0.674-0.675** | **0.680±0.037** | **0.680-0.680** | **0.676±0.031** | **0.676-0.677** |
| **Lasso** | **0.729±0.048** | **0.729-0.730** | **0.677±0.037** | **0.677-0.677** | **0.675±0.045** | **0.674-0.675** | **0.673±0.040** | **0.672-0.673** | **0.673±0.036** | **0.673-0.673** |
| **Not** | **0.731±0.039** | **0.731-0.731** | **0.678±0.029** | **0.677-0.678** | **0.674±0.039** | **0.674-0.675** | **0.677±0.027** | **0.676-0.677** | **0.675±0.027** | **0.675-0.675** |
| **P value** | **P<0.0001** | | **P<0.0001** | | **P<0.0001** | | **P<0.0001** | | **P<0.0001** | |

| **HbA1c** | **AUC** | | **Accuracy** | | **Precision** | | **Recall** | | **F1Score** | |
| --- | --- | --- | --- | --- | --- | --- | --- | --- | --- | --- |
|  | **Mean±SD** | **95%CI** | **Mean±SD** | **95%CI** | **Mean±SD** | **95%CI** | **Mean±SD** | **95%CI** | **Mean±SD** | **95%CI** |
| **Boruta** | **0.765±0.135** | **0.764-0.766** | **0.723±0.121** | **0.722-0.723** | **0.718±0.120** | **0.717-0.719** | **0.757±0.121** | **0.757-0.758** | **0.734±0.112** | **0.733-0.735** |
| **Lasso** | **0.772±0.131** | **0.772-0.773** | **0.720±0.129** | **0.720-0.721** | **0.720±0.127** | **0.720-0.721** | **0.758±0.132** | **0.757-0.759** | **0.733±0.116** | **0.733-0.734** |
| **Not** | **0.758±0.131** | **0.757-0.758** | **0.709±0.126** | **0.708-0.709** | **0.706±0.126** | **0.706-0.707** | **0.751±0.134** | **0.751-0.752** | **0.723±0.116** | **0.722-0.723** |
| **P value** | **P<0.0001** | | **P<0.0001** | | **P<0.0001** | | **P<0.0001** | | **P<0.0001** | |

**Table S8.Machine learning algorithm ten-fold cross-validation analysis results**

| **FBG** | | **AUC** | | | **Accuracy** | | | | | **Precision** | | | | **Recall** | | | **F1Score** | | | | |
| --- | --- | --- | --- | --- | --- | --- | --- | --- | --- | --- | --- | --- | --- | --- | --- | --- | --- | --- | --- | --- | --- |
|  |  | **Mean±SD** | **95%CI** | | **Mean±SD** | | | **95%CI** | | **Mean±SD** | | **95%CI** | | **Mean±SD** | | **95%CI** | **Mean±SD** | | **95%CI** | | |
| **AdaBoost** | | **0.741±0.015** | **0.738-0.744** | | **0.682±0.012** | | | **0.680-0.684** | | **0.679±0.019** | | **0.675-0.682** | | **0.677±0.020** | | **0.674-0.681** | **0.678±0.016** | | **0.675-0.681** | | |
| **Bagging** | | **0.747±0.033** | **0.741-0.753** | | **0.690±0.026** | | | **0.685-0.695** | | **0.694±0.038** | | **0.687-0.701** | | **0.668±0.026** | | **0.664-0.673** | **0.681±0.029** | | **0.675-0.686** | | |
| **Bernoulli_Naive_Bayes** | | **0.722±0.010** | **0.721-0.724** | | **0.673±0.008** | | | **0.672-0.675** | | **0.663±0.009** | | **0.661-0.665** | | **0.688±0.022** | | **0.684-0.692** | **0.675±0.015** | | **0.673-0.678** | | |
| **Decision_Tree** | | **0.738±0.019** | **0.735-0.742** | | **0.685±0.015** | | | **0.683-0.688** | | **0.689±0.037** | | **0.682-0.695** | | **0.671±0.038** | | **0.664-0.678** | **0.678±0.015** | | **0.675-0.681** | | |
| **Extra_Tree** | | **0.724±0.017** | **0.721-0.727** | | **0.677±0.013** | | | **0.674-0.679** | | **0.677±0.025** | | **0.673-0.682** | | **0.663±0.028** | | **0.658-0.668** | **0.669±0.016** | | **0.667-0.672** | | |
| **Gaussian_Naive_Bayes** | | **0.696±0.014** | **0.693-0.698** | | **0.647±0.012** | | | **0.645-0.649** | | **0.630±0.010** | | **0.628-0.631** | | **0.694±0.030** | | **0.689-0.700** | **0.660±0.017** | | **0.657-0.663** | | |
| **Gradient_Boosting** | | **0.750±0.018** | **0.747-0.753** | | **0.690±0.015** | | | **0.687-0.692** | | **0.695±0.029** | | **0.689-0.700** | | **0.666±0.017** | | **0.663-0.669** | **0.680±0.015** | | **0.677-0.682** | | |
| **LDA** | | **0.737±0.012** | **0.734-0.739** | | **0.678±0.007** | | | **0.676-0.679** | | **0.668±0.007** | | **0.666-0.669** | | **0.692±0.028** | | **0.687-0.697** | **0.679±0.016** | | **0.676-0.682** | | |
| **Logistic_Regression** | | **0.737±0.012** | **0.735-0.740** | | **0.679±0.008** | | | **0.677-0.680** | | **0.670±0.008** | | **0.669-0.672** | | **0.688±0.029** | | **0.683-0.693** | **0.679±0.017** | | **0.676-0.682** | | |
| **Multinomial_Naive_Bayes** | | **0.707±0.009** | **0.705-0.708** | | **0.666±0.006** | | | **0.665-0.667** | | **0.657±0.006** | | **0.656-0.658** | | **0.678±0.035** | | **0.672-0.684** | **0.667±0.017** | | **0.664-0.670** | | |
| **Passive_Aggressive** | | **0.610±0.052** | **0.600-0.619** | | **0.581±0.040** | | | **0.574-0.588** | | **0.575±0.044** | | **0.567-0.583** | | **0.579±0.076** | | **0.565-0.593** | **0.575±0.054** | | **0.565-0.585** | | |
| **QDA** | | **0.723±0.010** | **0.721-0.725** | | **0.670±0.008** | | | **0.669-0.672** | | **0.660±0.008** | | **0.659-0.662** | | **0.686±0.023** | | **0.682-0.690** | **0.673±0.015** | | **0.670-0.675** | | |
| **Random_Forest** | | **0.756±0.027** | **0.751-0.761** | | **0.696±0.022** | | | **0.692-0.700** | | **0.702±0.033** | | **0.696-0.708** | | **0.669±0.025** | | **0.664-0.674** | **0.685±0.025** | | **0.680-0.690** | | |
| **SGD** | | **0.736±0.012** | **0.734-0.739** | | **0.670±0.005** | | | **0.669-0.670** | | **0.652±0.004** | | **0.651-0.652** | | **0.712±0.034** | | **0.706-0.718** | **0.680±0.016** | | **0.677-0.683** | | |
| **XGBoost** | | **0.761±0.029** | **0.756-0.766** | | **0.698±0.023** | | | **0.694-0.702** | | **0.702±0.035** | | **0.696-0.708** | | **0.679±0.021** | | **0.675-0.683** | **0.690±0.024** | | **0.686-0.694** | | |
| **P value** | | **P<0.0001** | | | **P<0.0001** | | | | | **P<0.0001** | | | | **P<0.0001** | | | **P<0.0001** | | | | |
| **HbA1c** | | **AUC** | | | | | **Accuracy** | | | **Precision** | | | | **Recall** | | | | | **F1Score** | | |
|  |  | **Mean±SD** | | | **95%CI** | | **Mean±SD** | **95%CI** | | **Mean±SD** | | **95%CI** | | **Mean±SD** | | **95%CI** | | | **Mean±SD** | | **95%CI** |
| **AdaBoost** | | **0.782±0.141** | | | **0.756-0.807** | | **0.737±0.120** | **0.716-0.759** | | **0.762±0.130** | | **0.738-0.786** | | **0.737±0.150** | | **0.710-0.764** | | | **0.739±0.111** | | **0.719-0.759** |
| **Bagging** | | **0.796±0.161** | | | **0.767-0.825** | | **0.747±0.143** | **0.721-0.772** | | **0.763±0.131** | | **0.739-0.786** | | **0.741±0.145** | | **0.715-0.767** | | | **0.749±0.133** | | **0.725-0.773** |
| **Bernoulli_Naive_Bayes** | | **0.727±0.103** | | | **0.708-0.745** | | **0.682±0.101** | **0.664-0.701** | | **0.683±0.093** | | **0.666-0.700** | | **0.692±0.123** | | **0.670-0.715** | | | **0.685±0.100** | | **0.667-0.703** |
| **Decision_Tree** | | **0.773±0.142** | | | **0.747-0.799** | | **0.742±0.125** | **0.719-0.764** | | **0.750±0.133** | | **0.726-0.774** | | **0.773±0.101** | | **0.755-0.791** | | | **0.756±0.101** | | **0.738-0.775** |
| **Extra_Tree** | | **0.740±0.124** | | | **0.718-0.763** | | **0.710±0.104** | **0.691-0.729** | | **0.718±0.116** | | **0.697-0.739** | | **0.740±0.140** | | **0.715-0.765** | | | **0.719±0.095** | | **0.702-0.736** |
| **Gaussian_Naive_Bayes** | | **0.699±0.114** | | | **0.679-0.720** | | **0.606±0.114** | **0.585-0.626** | | **0.602±0.108** | | **0.582-0.622** | | **0.808±0.176** | | **0.776-0.840** | | | **0.671±0.090** | | **0.655-0.687** |
| **Gradient_Boosting** | | **0.801±0.151** | | | **0.774-0.829** | | **0.747±0.135** | **0.723-0.772** | | **0.765±0.131** | | **0.742-0.789** | | **0.754±0.130** | | **0.731-0.778** | | | **0.755±0.117** | | **0.734-0.776** |
| **LDA** | | **0.766±0.132** | | | **0.742-0.790** | | **0.716±0.118** | **0.695-0.737** | | **0.715±0.103** | | **0.696-0.734** | | **0.743±0.126** | | **0.720-0.765** | | | **0.726±0.106** | | **0.707-0.745** |
| **Logistic_Regression** | | **0.779±0.144** | | | **0.753-0.805** | | **0.743±0.129** | **0.720-0.767** | | **0.749±0.120** | | **0.727-0.771** | | **0.761±0.138** | | **0.736-0.786** | | | **0.751±0.118** | | **0.730-0.773** |
| **Multinomial_Naive_Bayes** | | **0.662±0.076** | | | **0.648-0.676** | | **0.603±0.054** | **0.593-0.613** | | **0.602±0.051** | | **0.592-0.611** | | **0.613±0.161** | | **0.584-0.642** | | | **0.599±0.086** | | **0.583-0.614** |
| **Passive_Aggressive** | | **0.694±0.148** | | | **0.668-0.721** | | **0.649±0.131** | **0.625-0.672** | | **0.646±0.148** | | **0.620-0.673** | | **0.648±0.186** | | **0.615-0.682** | | | **0.641±0.156** | | **0.612-0.669** |
| **QDA** | | **0.730±0.111** | | | **0.710-0.750** | | **0.680±0.100** | **0.662-0.698** | | **0.674±0.088** | | **0.658-0.690** | | **0.719±0.109** | | **0.699-0.738** | | | **0.694±0.090** | | **0.677-0.710** |
| **Random_Forest** | | **0.791±0.152** | | | **0.763-0.818** | | **0.740±0.135** | **0.715-0.764** | | **0.762±0.131** | | **0.738-0.786** | | **0.731±0.129** | | **0.708-0.754** | | | **0.743±0.121** | | **0.721-0.765** |
| **SGD** | | **0.785±0.140** | | | **0.760-0.810** | | **0.740±0.132** | **0.716-0.764** | | **0.744±0.125** | | **0.721-0.767** | | **0.767±0.127** | | **0.745-0.790** | | | **0.752±0.116** | | **0.731-0.773** |
| **XGBoost** | | **0.802±0.159** | | | **0.773-0.831** | | **0.753±0.146** | **0.727-0.779** | | **0.768±0.138** | | **0.743-0.793** | | **0.754±0.143** | | **0.728-0.780** | | | **0.758±0.134** | | **0.734-0.782** |
| **P value** | | **P<0.0001** | | | | | **P<0.0001** | | | **P<0.0001** | | | | **P<0.0001** | | | | | **P<0.0001** | | |

**Table S9. Machine learning bootstrapping sampling analysis results**

| **FBG** | **AUC** | | **Accuracy** | | **Precision** | | **Recall** | | **F1Score** | |
| --- | --- | --- | --- | --- | --- | --- | --- | --- | --- | --- |
|  | **Mean±SD** | **95%CI** | **Mean±SD** | **95%CI** | **Mean±SD** | **95%CI** | **Mean±SD** | **95%CI** | **Mean±SD** | **95%CI** |
| **AdaBoost** | **0.746±0.014** | **0.745-0.746** | **0.687±0.009** | **0.687-0.687** | **0.685±0.019** | **0.685-0.686** | **0.680±0.013** | **0.680-0.680** | **0.682±0.010** | **0.682-0.683** |
| **Bagging** | **0.751±0.033** | **0.751-0.752** | **0.693±0.025** | **0.693-0.694** | **0.698±0.037** | **0.698-0.699** | **0.670±0.022** | **0.670-0.670** | **0.684±0.027** | **0.683-0.684** |
| **Bernoulli_Naive_Bayes** | **0.725±0.010** | **0.725-0.725** | **0.677±0.007** | **0.677-0.677** | **0.668±0.011** | **0.668-0.668** | **0.689±0.016** | **0.689-0.689** | **0.678±0.013** | **0.678-0.678** |
| **Decision_Tree** | **0.742±0.019** | **0.742-0.742** | **0.689±0.014** | **0.688-0.689** | **0.690±0.030** | **0.690-0.690** | **0.675±0.032** | **0.675-0.675** | **0.682±0.018** | **0.681-0.682** |
| **Ensemble_Learning** | **0.767±0.029** | **0.767-0.768** | **0.705±0.023** | **0.705-0.706** | **0.711±0.038** | **0.710-0.711** | **0.683±0.014** | **0.683-0.683** | **0.696±0.023** | **0.696-0.696** |
| **Extra_Tree** | **0.726±0.018** | **0.726-0.727** | **0.680±0.016** | **0.679-0.680** | **0.685±0.028** | **0.685-0.685** | **0.654±0.022** | **0.654-0.654** | **0.669±0.015** | **0.668-0.669** |
| **Gaussian_Naive_Bayes** | **0.697±0.014** | **0.697-0.697** | **0.650±0.012** | **0.650-0.650** | **0.633±0.010** | **0.633-0.633** | **0.693±0.026** | **0.693-0.694** | **0.662±0.015** | **0.661-0.662** |
| **Gradient_Boosting** | **0.754±0.018** | **0.754-0.754** | **0.695±0.014** | **0.695-0.695** | **0.702±0.029** | **0.701-0.702** | **0.669±0.012** | **0.669-0.669** | **0.684±0.013** | **0.684-0.685** |
| **LDA** | **0.739±0.010** | **0.739-0.740** | **0.683±0.006** | **0.683-0.683** | **0.674±0.009** | **0.674-0.674** | **0.694±0.019** | **0.693-0.694** | **0.683±0.013** | **0.683-0.684** |
| **Logistic_Regression** | **0.740±0.011** | **0.740-0.740** | **0.683±0.006** | **0.683-0.683** | **0.675±0.010** | **0.675-0.675** | **0.689±0.020** | **0.689-0.690** | **0.682±0.014** | **0.682-0.682** |
| **Multinomial_Naive_Bayes** | **0.708±0.008** | **0.708-0.708** | **0.669±0.005** | **0.669-0.669** | **0.660±0.007** | **0.660-0.661** | **0.679±0.030** | **0.679-0.680** | **0.669±0.015** | **0.669-0.670** |
| **Passive_Aggressive** | **0.607±0.041** | **0.607-0.608** | **0.580±0.031** | **0.580-0.581** | **0.573±0.033** | **0.573-0.574** | **0.594±0.055** | **0.593-0.594** | **0.582±0.037** | **0.582-0.583** |
| **QDA** | **0.725±0.009** | **0.725-0.725** | **0.676±0.006** | **0.676-0.676** | **0.667±0.010** | **0.667-0.667** | **0.687±0.016** | **0.687-0.687** | **0.677±0.012** | **0.677-0.677** |
| **Random_Forest** | **0.760±0.027** | **0.760-0.760** | **0.701±0.021** | **0.700-0.701** | **0.708±0.034** | **0.708-0.709** | **0.672±0.018** | **0.672-0.672** | **0.689±0.023** | **0.689-0.690** |
| **SGD** | **0.740±0.011** | **0.740-0.740** | **0.674±0.004** | **0.674-0.674** | **0.656±0.005** | **0.656-0.656** | **0.715±0.026** | **0.715-0.715** | **0.684±0.013** | **0.684-0.684** |
| **XGBoost** | **0.764±0.028** | **0.763-0.764** | **0.701±0.023** | **0.701-0.701** | **0.706±0.036** | **0.706-0.706** | **0.679±0.017** | **0.679-0.679** | **0.692±0.024** | **0.692-0.692** |
| **P value** | **P<0.0001** | | **P<0.0001** | | **P<0.0001** | | **P<0.0001** | | **P<0.0001** | |

| **HbA1c** | **AUC** | | **Accuracy** | | **Precision** | | **Recall** | | **F1Score** | |
| --- | --- | --- | --- | --- | --- | --- | --- | --- | --- | --- |
|  | **Mean±SD** | **95%CI** | **Mean±SD** | **95%CI** | **Mean±SD** | **95%CI** | **Mean±SD** | **95%CI** | **Mean±SD** | **95%CI** |
| **AdaBoost** | **0.800±0.123** | **0.798-0.801** | **0.755±0.111** | **0.754-0.756** | **0.765±0.112** | **0.764-0.767** | **0.762±0.115** | **0.760-0.763** | **0.759±0.100** | **0.758-0.761** |
| **Bagging** | **0.799±0.152** | **0.797-0.801** | **0.751±0.133** | **0.749-0.753** | **0.754±0.128** | **0.752-0.755** | **0.770±0.129** | **0.768-0.772** | **0.759±0.123** | **0.758-0.761** |
| **Bernoulli_Naive_Bayes** | **0.733±0.082** | **0.732-0.734** | **0.696±0.082** | **0.695-0.697** | **0.682±0.078** | **0.681-0.683** | **0.736±0.104** | **0.735-0.737** | **0.706±0.085** | **0.705-0.707** |
| **Decision_Tree** | **0.776±0.136** | **0.774-0.778** | **0.747±0.118** | **0.746-0.749** | **0.748±0.121** | **0.746-0.749** | **0.786±0.095** | **0.784-0.787** | **0.762±0.097** | **0.761-0.763** |
| **Ensemble_Learning** | **0.843±0.118** | **0.841-0.844** | **0.784±0.120** | **0.783-0.786** | **0.784±0.120** | **0.783-0.786** | **0.806±0.106** | **0.805-0.807** | **0.793±0.108** | **0.792-0.795** |
| **Extra_Tree** | **0.746±0.107** | **0.745-0.748** | **0.712±0.104** | **0.711-0.714** | **0.715±0.121** | **0.713-0.716** | **0.751±0.109** | **0.750-0.753** | **0.725±0.091** | **0.724-0.727** |
| **Gaussian_Naive_Bayes** | **0.680±0.094** | **0.679-0.681** | **0.604±0.106** | **0.602-0.605** | **0.597±0.109** | **0.596-0.599** | **0.825±0.136** | **0.824-0.827** | **0.677±0.065** | **0.677-0.678** |
| **Gradient_Boosting** | **0.819±0.131** | **0.818-0.821** | **0.767±0.126** | **0.766-0.769** | **0.772±0.129** | **0.770-0.773** | **0.782±0.117** | **0.780-0.783** | **0.775±0.117** | **0.773-0.776** |
| **LDA** | **0.782±0.117** | **0.781-0.784** | **0.735±0.106** | **0.734-0.736** | **0.722±0.095** | **0.721-0.723** | **0.775±0.111** | **0.773-0.776** | **0.746±0.098** | **0.745-0.747** |
| **Logistic_Regression** | **0.797±0.128** | **0.795-0.799** | **0.754±0.126** | **0.752-0.755** | **0.748±0.119** | **0.747-0.750** | **0.782±0.123** | **0.781-0.784** | **0.763±0.118** | **0.762-0.765** |
| **Multinomial_Naive_Bayes** | **0.657±0.056** | **0.656-0.657** | **0.602±0.044** | **0.601-0.602** | **0.595±0.056** | **0.594-0.596** | **0.631±0.145** | **0.629-0.633** | **0.605±0.078** | **0.604-0.606** |
| **Passive_Aggressive** | **0.705±0.124** | **0.704-0.707** | **0.643±0.107** | **0.642-0.644** | **0.641±0.106** | **0.640-0.643** | **0.673±0.148** | **0.671-0.675** | **0.651±0.110** | **0.649-0.652** |
| **QDA** | **0.733±0.084** | **0.731-0.734** | **0.692±0.081** | **0.691-0.693** | **0.675±0.082** | **0.674-0.676** | **0.754±0.077** | **0.753-0.755** | **0.711±0.073** | **0.710-0.712** |
| **Random_Forest** | **0.811±0.132** | **0.810-0.813** | **0.753±0.131** | **0.751-0.755** | **0.763±0.126** | **0.761-0.764** | **0.764±0.131** | **0.762-0.765** | **0.759±0.119** | **0.758-0.761** |
| **SGD** | **0.776±0.164** | **0.774-0.778** | **0.735±0.153** | **0.733-0.737** | **0.730±0.131** | **0.728-0.732** | **0.779±0.128** | **0.778-0.781** | **0.752±0.127** | **0.751-0.754** |
| **XGBoost** | **0.813±0.142** | **0.811-0.815** | **0.763±0.135** | **0.761-0.765** | **0.768±0.128** | **0.766-0.769** | **0.773±0.131** | **0.772-0.775** | **0.769±0.126** | **0.767-0.771** |
| **P value** | **P<0.0001** | | **P<0.0001** | | **P<0.0001** | | **P<0.0001** | | **P<0.0001** | |

**Table S10 TRIPOD Checklist**

| **Section/Topic** | **Item** | **Checklist Item** | **Page** |
| --- | --- | --- | --- |
| **Title and abstract** | | | |
| Title | 1 | Identify the study as developing and/or validating a multivariable prediction model, the target population, and the outcome to be predicted. | 1 |
| Abstract | 2 | Provide a summary of objectives, study design, setting, participants, sample size, predictors, outcome, statistical analysis, results, and conclusions. | 1 |
| **Introduction** | | | |
| Background and objectives | 3a | Explain the medical context (including whether diagnostic or prognostic) and rationale for developing or validating the multivariable prediction model, including references to existing models. | 2-3 |
|  | 3b | Specify the objectives, including whether the study describes the development or validation of the model or both. | 2-3 |
| **Methods** | | | |
| Source of data | 4a | Describe the study design or source of data (e.g., randomized trial, cohort, or registry data), separately for the development and validation data sets, if applicable. | 3-4 |
|  | 4b | Specify the key study dates, including start of accrual; end of accrual; and, if applicable, end of follow-up. | Not applicable |
| Participants | 5a | Specify key elements of the study setting (e.g., primary care, secondary care, general population) including number and location of centres. | 3 |
|  | 5b | Describe eligibility criteria for participants. | 3 |
|  | 5c | Give details of treatments received, if relevant. | Not applicable |
| Outcome | 6a | Clearly define the outcome that is predicted by the prediction model, including how and when assessed. | 3 |
|  | 6b | Report any actions to blind assessment of the outcome to be predicted. | None |
| Predictors | 7a | Clearly define all predictors used in developing or validating the multivariable prediction model, including how and when they were measured. | Supplementary |
|  | 7b | Report any actions to blind assessment of predictors for the outcome and other predictors. | None |
| Sample size | 8 | Explain how the study size was arrived at. | 3 |
| Missing data | 9 | Describe how missing data were handled (e.g., complete-case analysis, single imputation, multiple imputation) with details of any imputation method. | 4 |
| Statistical analysis methods | 10a | Describe how predictors were handled in the analyses. | 4-5 |
|  | 10b | Specify type of model, all model-building procedures (including any predictor selection), and method for internal validation. | 4-5 |
|  | 10d | Specify all measures used to assess model performance and, if relevant, to compare multiple models. | 4-5 |
| Risk groups | 11 | Provide details on how risk groups were created, if done. | None |
| **Results** | | | |
| Participants | 13a | Describe the flow of participants through the study, including the number of participants with and without the outcome and, if applicable, a summary of the follow-up time. A diagram may be helpful. | 5-7 |
|  | 13b | Describe the characteristics of the participants (basic demographics, clinical features, available predictors), including the number of participants with missing data for predictors and outcome. | 5-7 |
| Model development | 14a | Specify the number of participants and outcome events in each analysis. | 5 |
|  | 14b | If done, report the unadjusted association between each candidate predictor and outcome. | 8-10 |
| Model specification | 15a | Present the full prediction model to allow predictions for individuals (i.e., all regression coefficients, and model intercept or baseline survival at a given time point). | None |
|  | 15b | Explain how to the use the prediction model. | 16 |
| Model performance | 16 | Report performance measures (with CIs) for the prediction model. | 10-11 |
| **Discussion** | | | |
| Limitations | 18 | Discuss any limitations of the study (such as nonrepresentative sample, few events per predictor, missing data). | 15 |
| Interpretation | 19b | Give an overall interpretation of the results, considering objectives, limitations, and results from similar studies, and other relevant evidence. | 12-14 |
| Implications | 20 | Discuss the potential clinical use of the model and implications for future research. | 15-16 |
| **Other information** | | | |
| Supplementary information | 21 | Provide information about the availability of supplementary resources, such as study protocol, Web calculator, and data sets. | 16 |
| Funding | 22 | Give the source of funding and the role of the funders for the present study. | 17 |

Application number：21ZDYF0656 Project number：2021YFS0197

**Sichuan Province Science and Technology Plan Project Contract**

Project type: Science and technology support plan

**project name: Construction of data-driven artificial intelligence prediction model/platform for treatment effect of type 2 diabetes and exploration of new model of individualized treatment**Organizer:Sichuan Academy of Medical Sciences·Sichuan Provincial People’s Hospital

Project manager：Enwu Long**Document date：2021-04-01~2023-03-31**

Approval Letter

| Project | **Construction of data-driven artificial intelligence prediction model/platform for treatment effect of type 2 diabetes and exploration of new model of individualized treatment** | | | |
| --- | --- | --- | --- | --- |
| Classification | **Clinical research** | **Research Type** | | **Non-intervention studies** |
| Review Categories | **Initial review** | **Review Pattern** | | **Quick review** |
| Department | **Department of Pharmacy** | **PI/Title** | | **Enwu Long/Deputy Chief Pharmacist** |
| **Form be handed** | | | | |
| **Clinical research protocol** | | | **Informed consent** | |
| **Researcher qualification** | | | **Other files** | |
| **Decision** | | | | |
| **Conclusion:agree** | | | | |
| **Comments:** | | | | |
| **According to the National Health and Family Planning Commission's "involves people of biomedical research ethics review method"(2016), the CFDA "quality control standard for clinical trials" (2003), "Standard for quality management of clinical trials on medical devices"(2016), "guiding principles for ethical review of clinical trials"(2010). WMA "the declaration of Helsinki "(2013) and CIOMS "the human body biomedical research international moral guide ethical principles" (2002), reviewed by this Ethics Committee, are as follows:**  **Please follow the protocol approved by the ethics committee to conduct clinical studies to protect subjects' health and rights.**  **Before the start of the research, please strictly follow the relevant provisions of the "Regulations of the People's Republic of China on the Administration of Human Genetic Resources" (State Order No. 717), apply to the Ministry of Science and Technology for approval or filing, and file with the Ethics Committee.**  **The review frequency is 12 months. Investigators are requested to submit the research progress report on time within one month before the deadline according to the follow-up review frequency. If serious adverse events occur, applicants are requested to submit serious adverse event reports and follow-up summary reports in a timely manner. When there is any situation that may significantly affect the research progress or increase the risk of the subjects, the applicant is requested to submit a written report to the ethics committee in a timely manner.**  **Subjects who did not meet the inclusion criteria or exclusion criteria were included in the study; subjects who met the requirements of the suspension test were not removed from the study; subjects were given wrong treatment or dose; In case of any possible adverse impact on the rights/health of the subject or the scientific nature of the study, the investigator shall submit a violation report.**  **If the applicant suspends or terminates the study in advance, please submit the suspension/termination report in time.**  **After completing the clinical study, the applicant is requested to submit a final report. If the project is not started within half a year from the date of approval, the approval document shall be automatically abolished, and the applicant shall re-submit the application for ethical review to the ethics committee.**  **2021.5.17** | | | | |


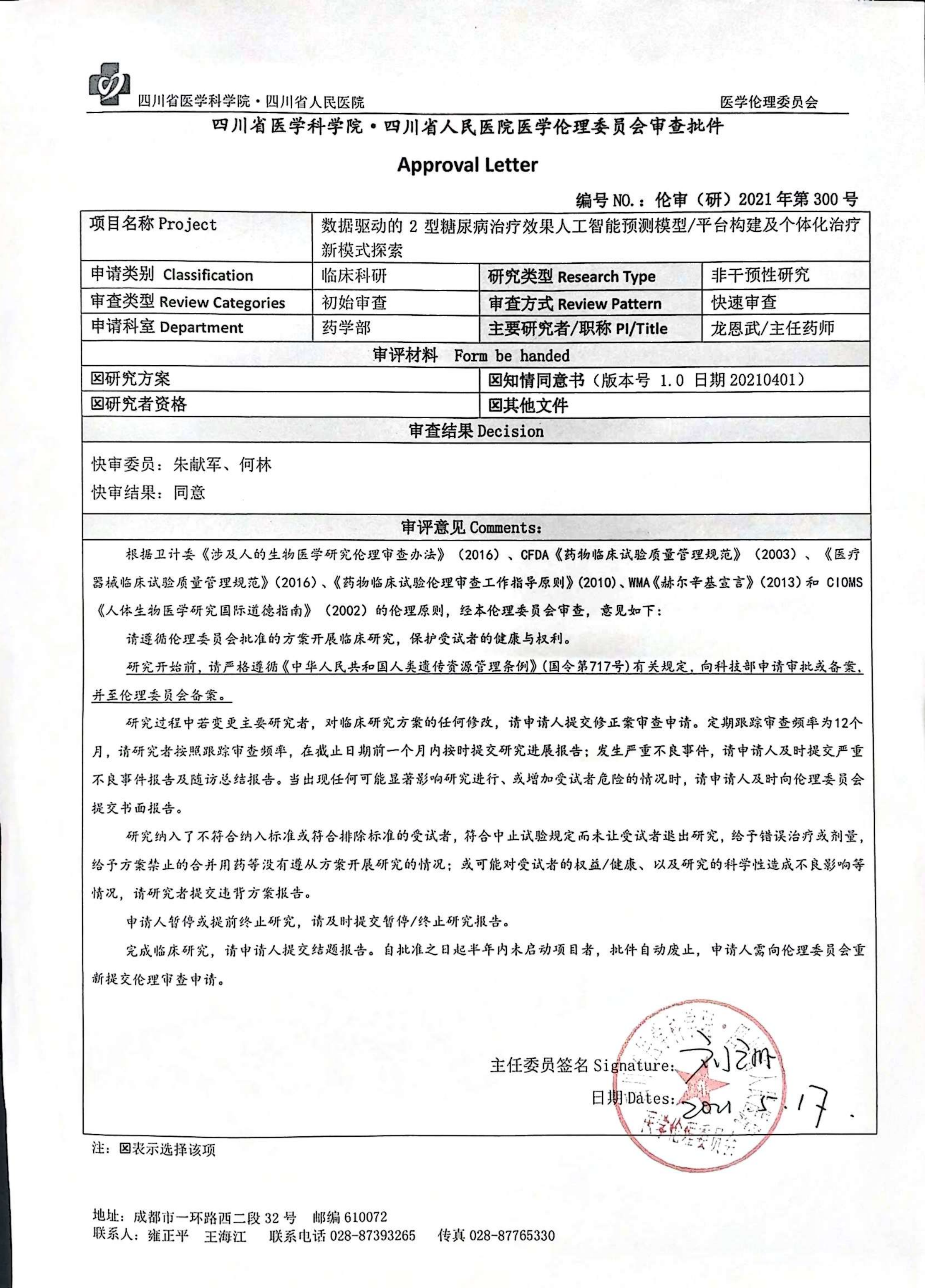


**Figure S1. Ethical approval document**


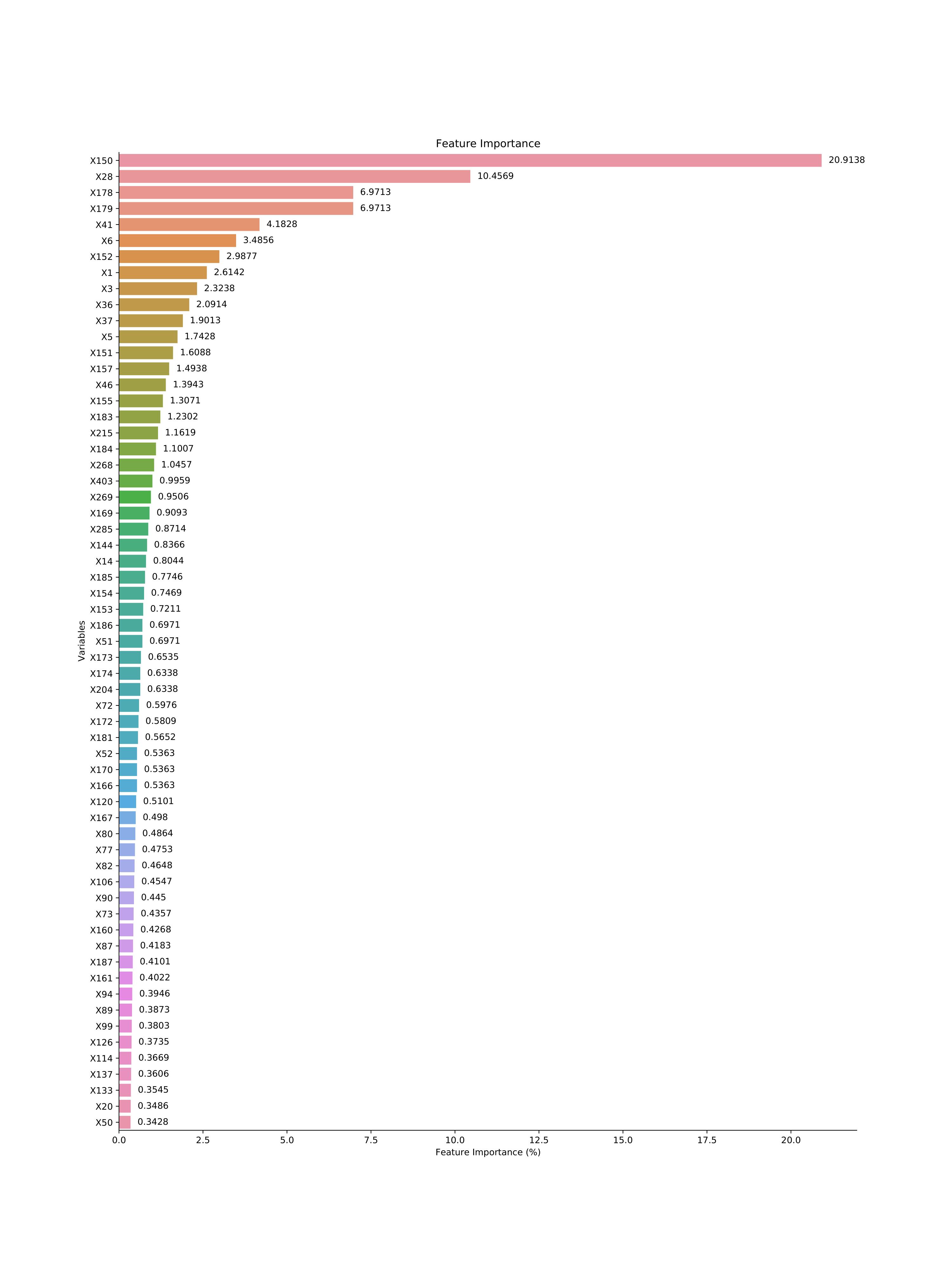


**Figure S2.FBG Feature importance bar chart (Imput method:Not;Screening method: Boruta )**


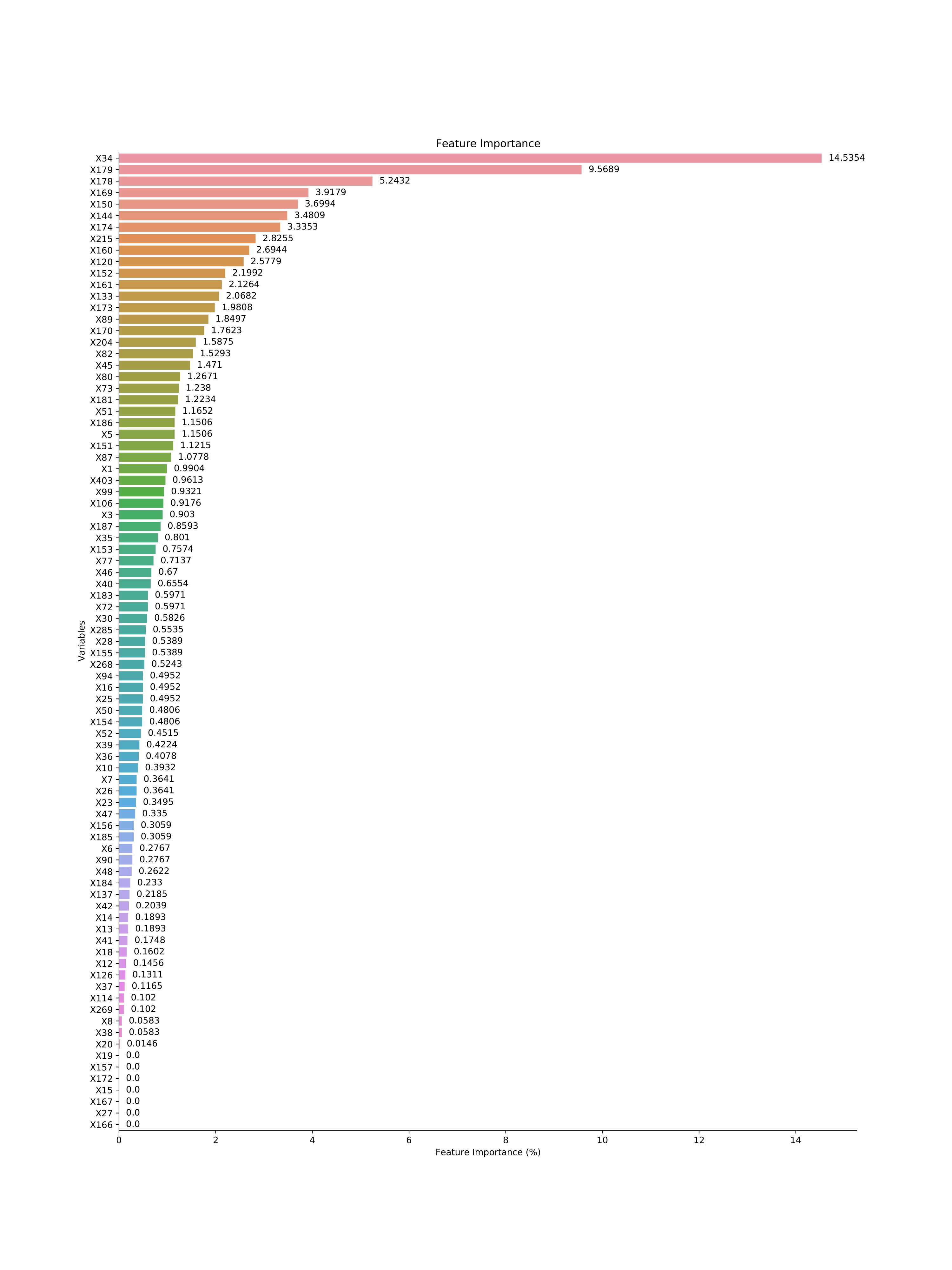


**Figure S3.FBG Feature importance bar chart (Imput method:Improved random**

**forest;Screening method: Lasso)**

**
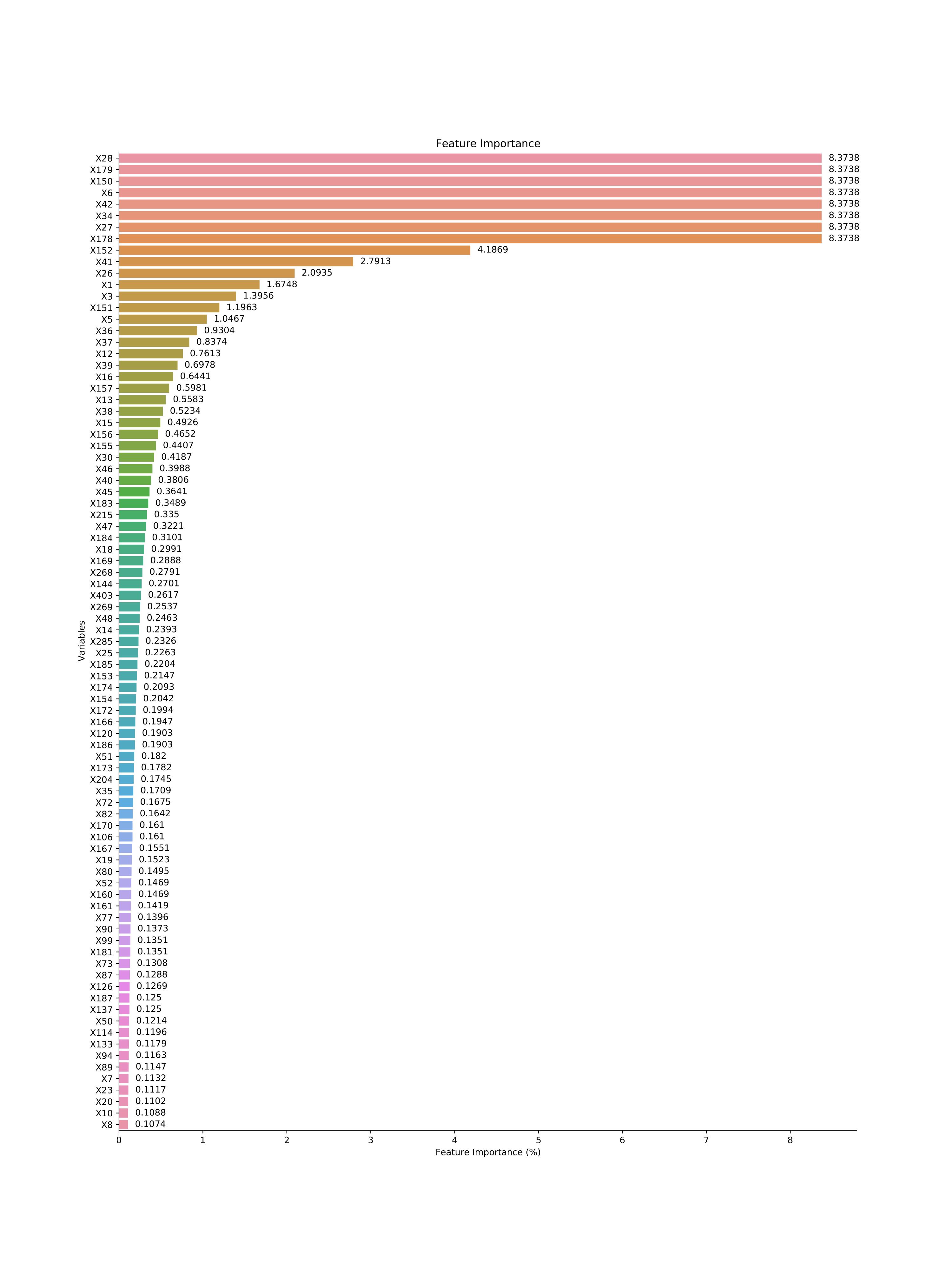
Figure S4.FBG Feature importance bar chart (Imput method:Improved random**

**forest;Screening method: Boruta)**


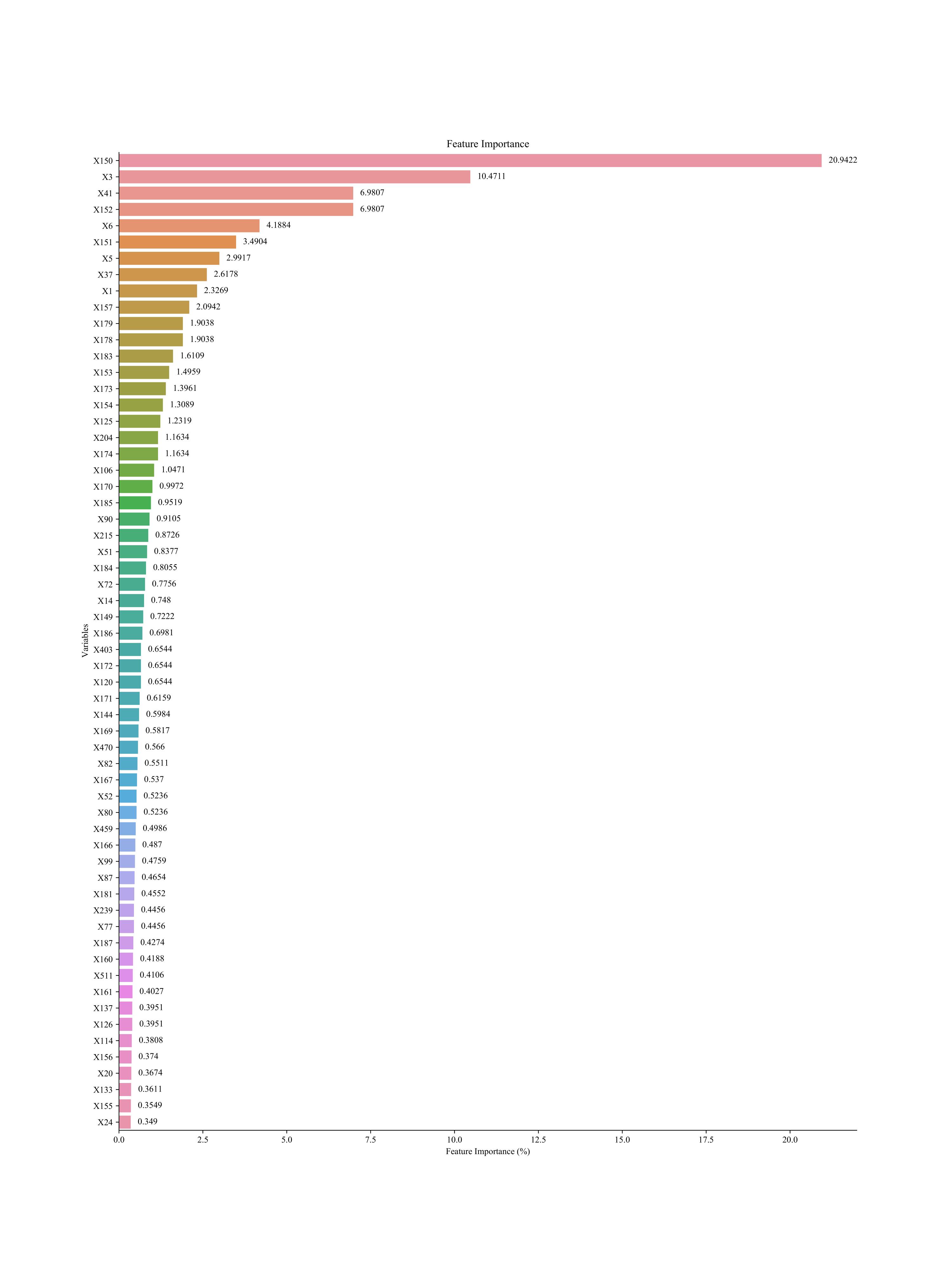


**Figure S5.HbA1c Feature importance bar chart (Imput method:Not;Screening method: Boruta )**


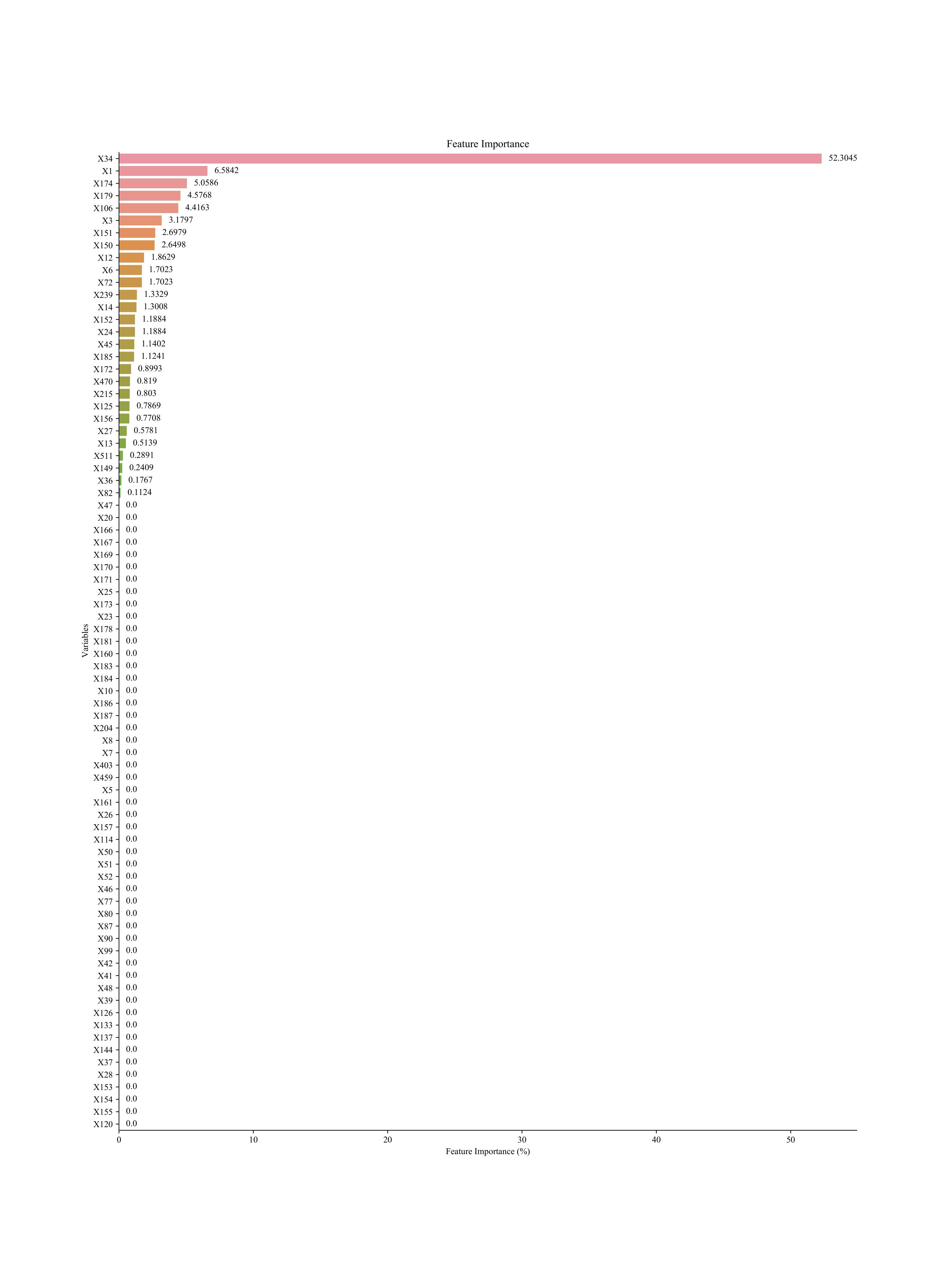
**Figure S6.HbA1c Feature importance bar chart (Imput method:Improved random**

**forest;Screening method: Lasso)**

**
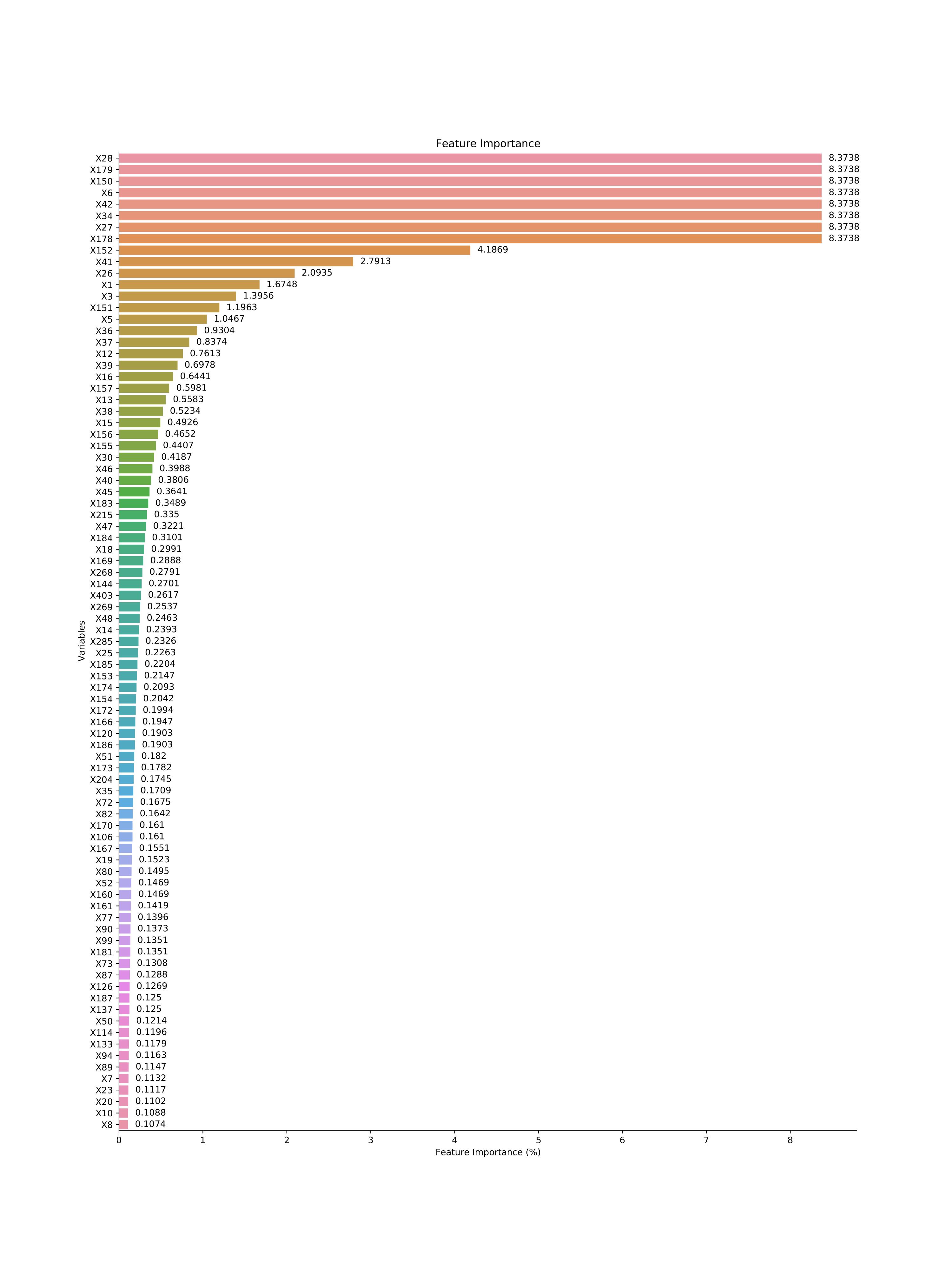
Figure S7.HbA1c Feature importance bar chart (Imput method:Improved random**

**forest;Screening method: Boruta)**
